# Tracking SARS-CoV-2 mutations and variants through the COG-UK-Mutation Explorer

**DOI:** 10.1093/ve/veac023

**Published:** 2022-03-18

**Authors:** Derek W Wright, William T Harvey, Joseph Hughes, MacGregor Cox, Thomas P Peacock, Rachel Colquhoun, Ben Jackson, Richard Orton, Morten Nielsen, Nienyun Sharon Hsu, The COVID-19 Genomics UK (COG-UK) consortium, Ewan M Harrison, Thushan I de Silva, Andrew Rambaut, Sharon J Peacock, David L Robertson, Alessandro M Carabelli

**Affiliations:** MRC-University of Glasgow Centre for Virus Research, University of Glasgow, Garscube Campus, 464 Bearsden Road, Glasgow G61 1QH, UK; MRC-University of Glasgow Centre for Virus Research, University of Glasgow, Garscube Campus, 464 Bearsden Road, Glasgow G61 1QH, UK; Department of Medicine, University of Cambridge, Addenbrookes Hospital, Hills Road, Cambridge CB2 0QQ, UK; Department of Infectious Disease, St Mary’s Medical School, Imperial College London, Praed Street, London, Westminster W2 1NY, UK; Institute of Evolutionary Biology, University of Edinburgh, Charlotte Auerbach Road, Edinburgh EH9 3FL, UK; MRC-University of Glasgow Centre for Virus Research, University of Glasgow, Garscube Campus, 464 Bearsden Road, Glasgow G61 1QH, UK; Institute of Evolutionary Biology, University of Edinburgh, Charlotte Auerbach Road, Edinburgh EH9 3FL, UK; MRC-University of Glasgow Centre for Virus Research, University of Glasgow, Garscube Campus, 464 Bearsden Road, Glasgow G61 1QH, UK; Department of Health Technology, Technical University of Denmark, Lyngby DK-2800, Denmark; The Florey Institute for Host-Pathogen Interactions and Department of Infection, Immunity and Cardiovascular Disease, Medical School, University of Sheffield, Beech Hill Road, Sheffield S10 2RX, UK; https://www.cogconsortium.uk,Full list of consortium names and affiliations are in; Department of Medicine, University of Cambridge, Addenbrookes Hospital, Hills Road, Cambridge CB2 0QQ, UK; Wellcome Sanger Institute, Wellcome Genome Campus, Hinxton CB10 1SA, UK; Department of Public Health and Primary Care, University of Cambridge, Worts Causeway, Cambridge CB1 8RN, UK; The Florey Institute for Host-Pathogen Interactions and Department of Infection, Immunity and Cardiovascular Disease, Medical School, University of Sheffield, Beech Hill Road, Sheffield S10 2RX, UK; Institute of Evolutionary Biology, University of Edinburgh, Charlotte Auerbach Road, Edinburgh EH9 3FL, UK; Department of Medicine, University of Cambridge, Addenbrookes Hospital, Hills Road, Cambridge CB2 0QQ, UK; MRC-University of Glasgow Centre for Virus Research, University of Glasgow, Garscube Campus, 464 Bearsden Road, Glasgow G61 1QH, UK; Department of Medicine, University of Cambridge, Addenbrookes Hospital, Hills Road, Cambridge CB2 0QQ, UK

**Keywords:** SARS-CoV-2, COVID-19, virus, spike, protein structure, antibody escape, antigenic variation, mutation, amino acid replacements, variants of concern, evasion, resistance, fitness, evolution

## Abstract

COG-UK Mutation Explorer (COG-UK-ME, https://sars2.cvr.gla.ac.uk/cog-uk/—last accessed date 16 March 2022) is a web resource that displays knowledge and analyses on SARS-CoV-2 virus genome mutations and variants circulating in the UK, with a focus on the observed amino acid replacements that have an antigenic role in the context of the human humoral and cellular immune response. This analysis is based on more than 2 million genome sequences (as of March 2022) for UK SARS-CoV-2 data held in the CLIMB-COVID centralised data environment. COG-UK-ME curates these data and displays analyses that are cross-referenced to experimental data collated from the primary literature. The aim is to track mutations of immunological importance that are accumulating in current variants of concern and variants of interest that could alter the neutralising activity of monoclonal antibodies (mAbs), convalescent sera, and vaccines. Changes in epitopes recognised by T cells, including those where reduced T cell binding has been demonstrated, are reported. Mutations that have been shown to confer SARS-CoV-2 resistance to antiviral drugs are also included. Using visualisation tools, COG-UK-ME also allows users to identify the emergence of variants carrying mutations that could decrease the neutralising activity of both mAbs present in therapeutic cocktails, e.g. Ronapreve. COG-UK-ME tracks changes in the frequency of combinations of mutations and brings together the curated literature on the impact of those mutations on various functional aspects of the virus and therapeutics. Given the unpredictable nature of SARS-CoV-2 as exemplified by yet another variant of concern, Omicron, continued surveillance of SARS-CoV-2 remains imperative to monitor virus evolution linked to the efficacy of therapeutics.

## 1. Introduction

As of March 2022, SARS-CoV-2, the causative agent of COVID-19, has accounted for over 450 million infections and 6 million deaths worldwide (https://covid19.who.int/). SARS-CoV-2 was first identified at the end of 2019 in the city of Wuhan, China, and has since spread with unprecedented efficiency among humans (Hu et al. 2021). In contrast to other RNA viruses, the *Coronaviridae* family is characterised by relatively high-replication fidelity due to the proofreading activity of their polymerases (Robson et al. 2020). Early analyses of SARS-CoV-2 genomes estimated an evolutionary rate of around 0.001 subs/site/year (two to three mutations per month) (Duchene et al. 2020); however, there is much deviation from this rate across the phylogeny with several outlier lineages,Appendix 1 including variants of concern (VOCs), that have rapidly acquired several mutations at a much higher rate than this. The analysis of mutations from virus genome data is important for basic virology (Houldcroft et al. 2017), to identify evolutionary signals associated with mutations prior to experimental and real-world data on clinical outcomes or vaccine effectiveness, and to document and track changes that could alter the effectiveness of therapeutics. At present, almost 9 million genome sequences are now available via the GISAID Initiative, permitting near real-time surveillance of the unfolding pandemic (Shu and McCauley 2017; Meredith et al. 2020).

SARS-CoV-2 showed relatively inconsequential genetic change until late 2020 (MacLean et al. 2021). Subsequently, later months of 2020 were characterised by the emergence, across the globe, of VOCs possessing mutations that altered virus phenotype in terms of transmissibility and antigenicity (Harvey et al. 2021). Concurrently, shifts in the immune profile of the human population likely represented a change in the selective environment evidenced by an increase in dN/dS ratios indicative at positive selection at codons across the genome and notable levels of convergence across the global phylogeny (Martin et al. 2021). The continuing emergence of SARS-CoV-2 variants exhibiting heightened transmissibility or antigenic novelty necessitates tools to detect, describe, and track those antigenic changes and make this information accessible to researchers, public health agencies, and drug and vaccine developers so that the information becomes actionable.

Since the beginning of the pandemic, several bioinformatics tools have been developed to analyse and generate outputs that support actionable information (e.g. Pangolin lineages https://cov-lineages.org/index.html; https://filogeneti.ca/covizu/; https://outbreak.info; COVID-19 CG https://covidcg.org; https://coval.ccpem.ac.uk/; CoV-GLUE http://cov-glue.cvr.gla.ac.uk, https://nextstrain.org; and https://covariants.org—last accessed date: 16 March 2022). Although these tools have been essential for data curation, analysis research, and public health impact (Hufsky et al. 2021), they have been mainly focusing on the epidemiological aspects of the pandemic, lacking the relevant information from the literature on the immunological effect of mutations.

This scientific need led us to create the COG-UK-Mutation Explorer (COG-UK-ME), a web resource that provides tracking of non-synonymous mutations in SARS-CoV-2 genome. COG-UK-ME is based on UK data, and it has been developed by the COVID-19 Genomics UK (COG-UK) consortium—created to deliver large-scale and rapid whole-genome virus sequencing to local National Health Service centres and the UK government. COG-UK-ME relies on CLIMB-COVID, a data-centric bioinformatics environment for centralising UK SARS-CoV-2 sequences (Nicholls et al. 2021a). Here, we describe COG-UK-ME and its main functionality. COG-UK-ME currently has around 5,000 users per month, with approximately 30 per cent from the UK, 20 per cent from the USA, and the remainder from other international locations.

COG-UK-ME has three aims: first, to make available amino acid mutations in a user-friendly way, enabling data transparency; second, to report on amino acid variation present in SARS-CoV-2 sequences that have been shown to confer resistance against antibodies or disrupt T cell epitope binding. The third is to report on the emergence of new mutations that have the potential to reduce the effectiveness of some therapeutics that have been granted approval for use. Data accumulating over a time course can be analysed so that trends can be detected and tracked.

## 2. Data analysis

COG-UK-ME is a publicly accessible web resource that displays in-depth information and analyses of SARS-COV-2 virus genome mutations and variants. Sequence information is deposited daily on the MRC CLIMB-COVID platform (Nicholls et al. 2021a), which has been generated by the COG-UK Consortium, Wellcome Sanger Institute, public health agencies, and other approved providers. Virus lineages are assigned by using a phylogenetic framework to identify those lineages that contribute most to active spread (Rambaut et al. 2020; O’Toole et al. 2021). Mutations for UK sequences are then analysed on the CLIMB platform and linked with curated data on antigenicity, therapeutics, and drug resistance. The prepared data files are then transferred from CLIMB to a web server and visualised.

### 2.1 Tracking changes in the mutation count

**Figure 1.**
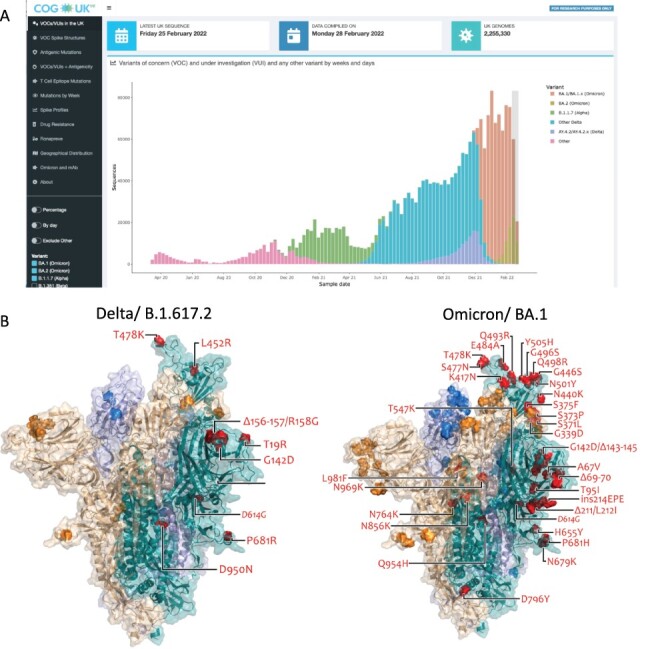
(A) Frequency plot showing the number of SARS-CoV-2 sequences per week for VOCs Alpha, Delta, Delta-AY4.2, Omicron BA.1 and BA.2, and ‘other’ pre-VOC variants (see key) in the UK. The light grey box covering the two most recent weeks indicates a period in which sequence counts are low due to a lag (Figure S1). (B) Spike protein structure showing locations of Delta- and Omicron-specific spike mutations. Ectodomain of the spike homotrimer in open conformation with individual spike protein chains shown in different colours. On each monomer, highlighted spheres show the locations of amino acid substitutions, deletions (Δ), or insertions (ins) that distinguish the Omicron (BA.1) variant, relative to the original genotype (Wuhan-Hu-1). These are annotated on the monomer with an ‘up’ receptor-binding domain where they are highlighted in red on teal. The substitution D614G, which is shared by common descent by all lineage B.1 descendants is italicised. The visualisation is made using a complete spike model (Woo et al. 2020), which is in turn based upon a partial cryo-EM structure (RCSB Protein Data Bank (PDB) ID: 6VSB (Wrapp et al. 2020)).

COG-UK-ME shows a browsable dataset of all the amino acid sequence variations in SARS-CoV-2 protein sequences. These are shown for all data, and in the recent past—over the last 28 days—in the UK and in the four UK nations (England, Scotland, Wales, and Northern Ireland) (‘Mutation Counts’ and ‘Mutations by week’ tabs). The ‘VOCs and VUIs in the UK’ tab shows through tables and visualisations the number of sequences of variants under investigation (VUI) and VOCs as designated by the UK Health Security Agency (formerly Public Health England) (https://www.gov.uk/government/publications/covid-19-variants-genomically-confirmed-case-numbers/variants-distribution-of-cases-data—last accessed date: 16 March 2022) (Fig. 1A). COG-UK-ME also provides visualisations of the spike protein structure showing the position of the VOC-defining mutations (Fig. 1B). Data are also placed in their geographical context by showing the number of sequences and percentage of variants per region (Nomenclature of Territorial Units for Statistics—NUTS1) (‘Geographical distribution’ tab).

### 2.2 Spike profile tracking

**Figure 2.**
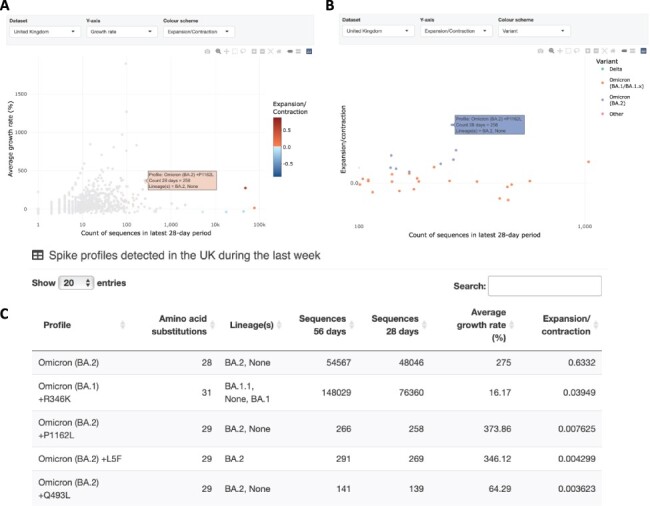
Spike profiles sampled within 7 days of the latest UK sequence are summarised. Each spike profile is a set of amino acid substitutions listed relative to the original genotype (Wuhan-Hu-1). Figure prepared with data compiled on 27 February 2022 with a most recent sequence date of 24 February 2022. A) Points represent spike profiles positioned by the number of sequences in the latest 28-day period and the average growth rate calculated over the latest 56-day period. Points are coloured by an expansion/contraction statistic that takes both the rate of change in frequency and the overall frequencies of a profile into account. The cursor is hovering to show information associated with BA.2 + P1162L. B) Points represent spike profiles positioned by the number of sequences in the latest 28-day period and the expansion/contraction statistic used to colour points in **A**. Here, points are coloured to show profiles associated with Delta, Omicron (BA.1/BA.1.x), and Omicron (BA.2) variants. The cursor is again positioned to highlight the position of BA.2 + P1162L. The plot has been zoomed to focus on profiles with 28-day counts between 100 and 1,000. C) Searchable table sorted to show the four profiles in the UK with the highest values in the expansion/contraction column. Further columns show profile numbers in the latest 28- and 56-day periods and average growth rate.

In addition to tracking the frequency of individual substitutions across the genome and of lineages identified as VOCs or VUIs, changes in the frequency of combinations of spike amino acid substitutions are tracked. Each spike profile is defined as the combination of substitutions compared with the original genotype (Wuhan-Hu-1). Profiles may represent monophyletic lineages or they may have arisen convergently across the phylogeny. Changes in profile frequency over the latest 56-day period are considered. For currently circulating profiles (those sampled within the latest 7 days), a sortable and searchable table includes information on the pango lineage(s) for which the profile has been associated, the number of substitutions comprising the profile, and the count of sequences across the latest 56-day and 28-day periods. The average growth rate (plotted on the y-axis in Fig. 2A) is calculated as the mean percentage change in frequency between each 2-week period within the 56-day period. As growth rates are sensitive to potentially stochastic changes at very low frequencies, we also calculate a statistic that estimates recent expansion or contraction of each profile, calculated over the 56-day period (plotted on the y-axis in Fig. 2B). For each profile, $i$, the absolute value for this statistic, ${X_i}$, is calculated using the observed frequency, ${O_{i,j}}$, of each profile, $i$, in each of the most recent 2-week periods, $j$, according to

$${X_i} = \mathop \sum \nolimits {{{{\left( {{O_{i,j}} - {E_i}} \right)}^2}} \over {{E_i}}}{6pt}$$

where ${E_i}$ is the frequency of profile $i$ over the full 8-week period under consideration. Thus, the value calculated is influenced by both the rate of change in profile frequency and the overall frequencies of a given profile and is more robust to stochastic differences in profile frequency that tend to occur at low frequencies.

This monitoring of spike profiles allows the detection of emerging, potentially advantageous, spikes that might not be detected by surveillance methods conditioned on mutations previously determined to be noteworthy through experimentation or other means. This simple approach is complementary to more sophisticated phylogenetic approaches for the estimation of lineage-specific growth rates. One advantage of this simple non-phylogenetic approach is that the convergent accumulation of a substitution or combination of substitutions on a particular background is identified. Such a scenario could arise when there is strong selective pressure on a genotype (e.g. the introduction of a therapeutic). For example, this approach would quickly alert to the growth of a profile such as Delta + E484K emerging convergently across the Delta phylogeny in response to within-host, immune-mediated selection, even if the instances of this profile are interspersed across the phylogeny.

### 2.3 Antigenic changes

**Figure 3.**
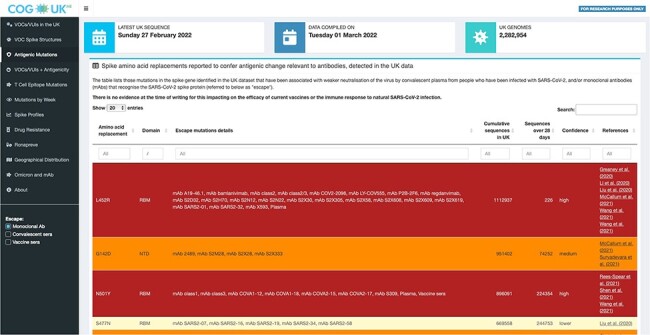
Amino acid substitutions in the spike protein identified in the UK dataset (referred to as ‘Escape mutations’) that have been associated with weaker neutralisation of the virus by convalescent or post-vaccination plasma/serum or spike-specific monoclonal antibodies (mAbs) or that have been observed to emerge upon exposure to either mAbs or plasma in laboratory experiments.

**Figure 4.**
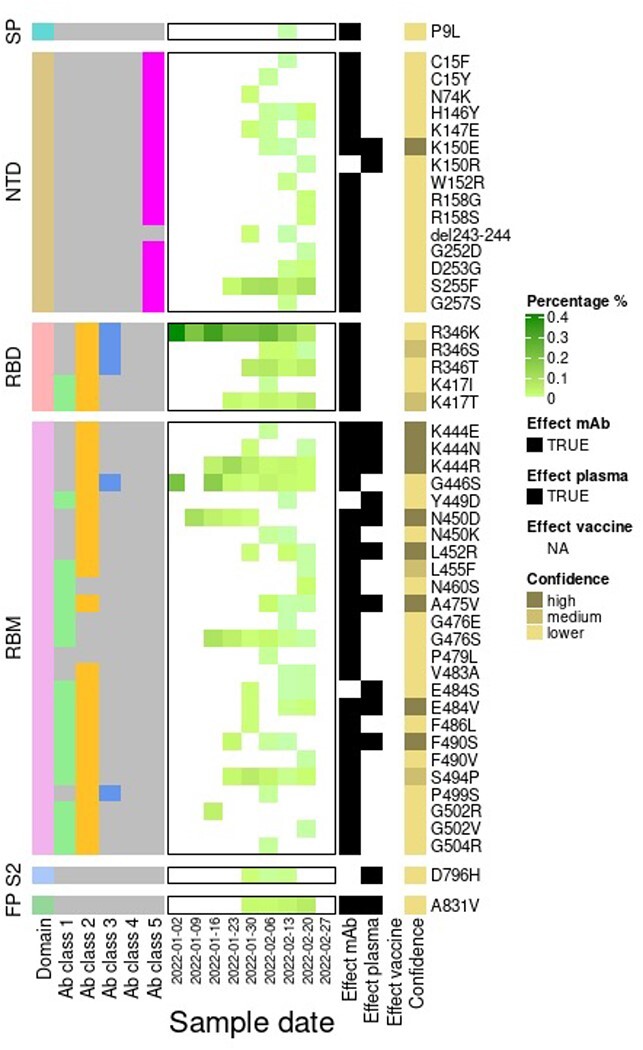
Heatmap showing the frequency of spike amino acid substitutions and a deletion with a potential or confirmed antigenic role on top of BA.2 through time. The labelled structural domains are indicated on the left side: SP, signal peptide; NTD, N-terminal domain; RBD, receptor-binding domain; RBM, receptor-binding motif; S2, subunit; FP, fusion peptide. Residues are also coloured according to the class of antibody that binds to an epitope. RBD antibody Classes 1–4 (Barnes et al. 2020) are depicted by colours: green (Class 1: ACE2 blocking, bind open RBD only), yellow (Class 2: ACE2 blocking, bind open, and closed RBD), blue (Class 3: non-ACE2 blocking, bind open, and closed RBD), or yellow (Class 4: non-ACE2 blocking, bind open RBD only). Residues described in an NTD epitope (Chi et al. 2020) are coloured in magenta (Class 5). Each residue is also classified as having evidence for mutations either affecting neutralisation by mAbs (Baum et al. 2020; Li et al. 2020; Weisblum et al. 2020; Liu et al. 2021) or serum from previously infected individuals (convalescent plasma) (Li et al. 2020; Weisblum et al. 2020; Andreano et al. 2021; Greaney et al. 2021; Liu et al. 2021) or vaccinated individuals (Wang et al. 2021) and emerging upon exposure to mAbs (Baum et al. 2020; Weisblum et al. 2020; Liu et al. 2021) or plasma (Weisblum et al. 2020; Andreano et al. 2021) in laboratory experiments.

The ‘Antigenic changes’ tab shows a table listing all mutations in the spike protein present in the UK sequence dataset that have individually been associated with some significant degree of weaker virus neutralisation by convalescent plasma, post-vaccination sera, or SARS-CoV-2 spike-specific mAbs (referred to as ‘Escape mutations’ in Fig. 3). Alongside links to the associated literature for each substitution, a confidence score representing the weight of evidence associated with each substitution is shown: ‘high’, whenever the antigenic role of mutation is supported by multiple studies, including at least one that reports an effect observed with (post-infection serum) convalescent plasma; ‘medium’, if the antigenic role of the mutation is supported by multiple studies; and ‘low’, when the mutation is supported by a single study (Fig. 3). In the ‘VOCs + Antigenicity’ tab, COG-UK-ME reports the occurrence of additional amino acid substitutions or deletions linked to antigenic change within each VOC (Fig. 4). Relative proportions (expressed as percentages) of sequences carrying specific mutations can give information about the antigenic diversity within a VOC lineage.

*High confidence (red) refers to an antigenic role supported by multiple studies, including at least one that reports an effect observed with (post-infection serum) convalescent plasma; ‘medium’ (orange), if the antigenic role is supported by multiple studies; and ‘low’ (yellow), when the mutation is supported by a single study. The boxes above the table enable filtering by multiple criteria.

### 2.4 T cell epitope mutations

Similar to the ‘Antigenic changes’ tab, the ‘T cell epitope mutations’ tab shows amino acid replacements in experimentally proven T cell epitopes both in spike and in other proteins, which have been described in the literature. Data are further filtered based on experimental studies just defining T cell epitopes (‘Epitope studies’) or those reporting on the impact of specific mutations on T cell recognition (‘Reduced T cell recognition’). Also shown are predicted antigen presentation likelihood percentile rank values to the experimentally proposed HLA restriction element based on the NetMHCpan (CD8) and NetMHCIIpan (CD4) 4.1 algorithms (https://services.healthtech.dtu.dk/service.php?NetMHCpan-4.1 and https://services.healthtech.dtu.dk/service.php?NetMHCIIpan-4.0—last accessed date: 16 March 2022) (Reynisson et al. 2020) for both the wild-type and mutant peptide variants. Here, peptides with predicted percentile rank scores of less than 2.0 for CD8 and less than 5.0 for CD4 are likely HLA binders. Amino acid replacements in any epitope are visualised through logo plots, in which each letter represents an amino acid replacement present in a specific epitope, and its height represents residue frequency. The number below the sequence logo shows the position relative to the start position of the epitope.

### 2.5 Drug resistance

**Figure 5.**
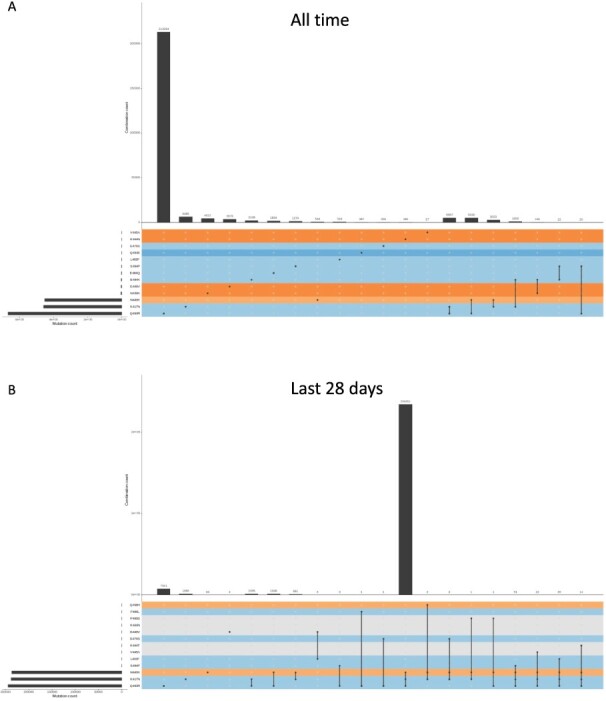
UpSet plot showing the counts of mutations affecting Ronapreve constituent mAbs that have occurred individually and in combinations (Lex et al. 2014). Occurrence is shown in the full UK SARS-CoV-2 genome sequence dataset (A) and in a dataset compiled of sequences in the latest 28-day period (B). Spike amino acid substitutions known to affect either casirivimab or imdevimab mAbs were considered. The upper histogram shows the number of sequences per mutation (dots) or combination of mutations (lines), and the bottom left histogram presents the number of sequences with each specific substitution. Rows are coloured according to the mAb to which the greatest fold decrease in binding was recorded (blue = casirivimab, orange = imdevimab), with a lighter shade indicating a fold decrease of less than 100 and darker shade indicating 100 or greater.

The ‘Drug resistance’ and ‘Ronapreve’ tabs show tables and visualisations for those mutations associated with the resistance of SARS-CoV-2 to antiviral treatments (e.g. Remdesivir) and therapeutic mAb cocktails that are currently used in clinical settings (e.g. Ronapreve, cocktail of *casirivimab* and *imdevimab*) (Beigel et al. 2020; Sidebottom and Gill 2021). The UpSet plot in the Ronapreve tab allows users to track amino acid substitutions known to affect either *casirivimab* or *imdevimab* mAbs and in combination (Fig. 5). Other therapeutics will be added in the future.

## 3. Concluding remarks

Bioinformatics resources such as COG-UK-ME play an important role by providing clear and accessible information to those who are tackling the pandemic, including through public health actions and the development of vaccines and therapeutics. COG-UK-ME is unique in presenting data from a densely sequenced population with an emphasis on publicly available data (bioproject accession PRJEB37886 and public alignments https://www.cogconsortium.uk/tools-analysis/public-data-analysis-2/—last accessed date: 16 March 2022). COG-UK-ME also brings together curated literature on the impact of mutations on various functional aspects of the virus. The COG-UK-ME interface allows users to track mutations that are a potential threat based on a phenotypic impact on virus biology or by conferring resistance to the human immune response, including that boosted by vaccines or antiviral drugs. Rapid analyses of VOCs, e.g. the accumulation of any mutation, can also be obtained from the COG-UK-ME interface. Of particular interest to researchers and for therapeutics are mutations that either have an antigenic role or affect T cell binding. These mutations are intensely monitored by researchers and Public Health Agencies to identify any new variant that could escape the immunity generated by vaccines. Timely identification of VOC/VUI samples can facilitate access to clinical specimens to isolate live virus and serum for further immunological evaluation.

Although amino acid sequence analyses are not sufficient to determine the functional effect of a single mutation on SARS-CoV-2 fitness when taken in isolation, COG-UK-ME strives to collate all the available literature on SARS-CoV-2 mutations and provides data to support experiments that investigate the change in phenotype that these mutations might confer on variants.

## 4. Methods

Throughout COG-UK-ME, Wuhan-Hu-1 (NCBI RefSeq NC_045512) is used as the reference sequence for nucleotide coordinates, codon numbering within viral proteins, and wild-type amino acid assignments. Sequences are regularly uploaded onto the MRC-CLIMB platform. Sequences with quality issues are excluded. Amino acid replacements and in-frame indels in each sequence are identified (Nicholls et al. 2021a).

Source code is available at https://github.com/wrightdw/COG-UK-ME (last accessed date: 16 March 2022).

### 4.1 Data preparation

Sequence metadata files are processed on the CLIMB-COVID platform (Nicholls et al. 2021a) using the R statistical programming language (Team 2021) and the Tidyverse collection of R packages (Wickham et al. 2019). Non-UK sequences are filtered out. Amino acid replacements and reference amino acids are counted for all times and for a 28-day period up to and including the latest sequence date for the UK and the four UK nations. Counts are linked with data on antigenic changes, data on therapeutics, epitope data and predicted epitope binding percentile rank values. Counts of all amino acids across all positions in the spike protein are prepared for the visualisation of sequence logos.

PANGO lineage name aliases are resolved to the full lineage names using the current designations at https://github.com/cov-lineages/pango-designation (last accessed date: 16 March 2022). VOC and VUI lineages are counted by day and by week for the UK and the four UK nations, counting AY.x sub-lineages within the Delta VOC hierarchically. VOC lineages are also counted by week and by geographic region according to the 12 Nomenclature of Territorial Units for Statistics first-level regions of the UK (NUTS1). Antigenic amino acid replacements and deletions in the spike protein are counted for VOC lineages, excluding lineage defining replacements, as defined by the UK Health Security Agency at https://github.com/phe-genomics/variant_definitions (last accessed date: 16 March 2022). Following data preparation, the resultant data files are transferred from CLIMB to a web server for visualisation.

### 4.2 Literature search

We searched PubMed, LitCovid, BioRxiv, and MedRxiv using the search term ‘SARS-CoV-2’ combined with ‘mAbs’, ‘monoclonal’, ‘convalescent’, ‘neutralisation/neutralization’, ‘epitope’, and ‘antibody’ for studies published from January 2020 to July 2021 and manually searched the references of select articles for additional relevant articles (Figure S2). We also searched BioRxiv, and MedRxiv using combinations of the search terms: ‘COVID19’, ‘COVID-19’, ‘SARS-CoV-2’, ‘remdesivir’, ‘favipiravir’, ‘molnupiravir’, ‘nirmatrelvir’, ‘ritonavir’, ‘paxlovid’, ‘antiviral’, ‘binding’, ‘efficacy’, ‘effective’, ‘resistance’, ‘resistant’, ‘sensitivity’, ‘inhibit’, ‘evasion’, ‘mutation’, and ‘variant’. Results reporting on SARS-CoV-2 mutations that cause resistance to antiviral drugs were recorded and published on the dashboard. This included many different types of assays and studies: neutralisation assays, receptor binding assays, clinical efficacy studies, transcriptional inhibition assays, and in silico indications of resistance. Antiviral drugs are included in the review if they are clinically approved somewhere in the world or are in Stage 3 clinical trials. This search is repeated each week, allowing the timely updating of the dashboard when new research arises.

### 4.3 Data visualisation

The Shiny framework is used to create the COG-UK-ME web application, hosted in the Shiny Server environment (Chang et al. 2021). In order to maximise performance across multiple concurrent users, most values are pre-computed in the data preparation process on CLIMB, with the web application focussing on data visualisation.

The bar charts for VOC lineages and mutations, the geographical maps of VOC lineages, and the scatter plot of spike profiles are generated using *ggplot2*, with interactive features added using *Plotly* (2015). The heatmap of antigenic changes in the spike protein is generated using the *ComplexHeatmap* package (Gu et al. 2016), antigenic replacements, and structural domain classifications. Amino acid replacements in epitopes are visualised as sequence logos using the *ggseqlogo* package (Wagih 2017). UpSet plots for mutations affecting Ronapreve are generated using the *UpsetR* package (Lex et al. 2014; Conway et al. 2017). The web application user interface is created using the *shinydashboard* (Chang and Ribeiro 2021), *shinydashboardPlus* (Granjon 2021), *shinyWidgets* (Perrier et al. 2021), and *shinyjs* packages (Attali 2020).

For the visualisations of the VOC spike mutations on the structure, the file 6vsb_1_1_1.pdb containing a complete model of the full-length glycosylated spike homotrimer in open conformation with one monomer having the receptor-binding domain in the ‘up’ position was obtained from the CHARMM-GUI Archive (Woo et al. 2020; CHARMM-GUI Archive, 2021). This model is itself generated based upon a partial spike cryo-EM structure (PDB ID: 6VSB (Wrapp et al. 2020)). For visualisation, the model was trimmed to the ectodomain (Residues 14–1164) and the signal peptide (Residues 1–13) and glycans were removed. Figures were prepared using PyMol (Schrödinger-LLC 2010).

## Supplementary Material

veac023_Supp

## Appendix

### The COVID-19 Genomics UK (COG-UK) consortium June 2021 V.1

**Funding acquisition, Leadership and supervision, Metadata curation, Project administration, Samples and logistics, Sequencing and analysis, Software and analysis tools, and Visualisation:**

Dr Samuel C Robson ^13, 84^

**Funding acquisition, Leadership and supervision, Metadata curation, Project administration, Samples and logistics, Sequencing and analysis, and Software and analysis tools:**

Dr Thomas R Connor ^11, 74^ and Prof Nicholas J Loman ^43^

**Leadership and supervision, Metadata curation, Project administration, Samples and logistics, Sequencing and analysis, Software and analysis tools, and Visualisation:**

Dr Tanya Golubchik ^5^

**Funding acquisition, Leadership and supervision, Metadata curation, Samples and logistics, Sequencing and analysis, and Visualisation:**

Dr Rocio T Martinez Nunez ^46^

**Funding acquisition, Leadership and supervision, Project administration, Samples and logistics, Sequencing and analysis, and Software and analysis tools:**

Dr David Bonsall ^5^

**Funding acquisition, Leadership and supervision, Project administration, Sequencing and analysis, Software and analysis tools, and Visualisation:**

Prof Andrew Rambaut ^104^

**Funding acquisition, Metadata curation, Project administration, Samples and logistics, Sequencing and analysis, and Software and analysis tools:**

Dr Luke B Snell ^12^

**Leadership and supervision, Metadata curation, Project administration, Samples and logistics, Software and analysis tools, and Visualisation:**

Rich Livett ^116^

**Funding acquisition, Leadership and supervision, Metadata curation, Project administration, and Samples and logistics:**

Dr Catherine Ludden ^20, 70^

**Funding acquisition, Leadership and supervision, Metadata curation, Samples and logistics, and Sequencing and analysis:**

Dr Sally Corden ^74^ and Dr Eleni Nastouli ^96, 95, 30^

**Funding acquisition, Leadership and supervision, Metadata curation, Sequencing and analysis, and Software and analysis tools:**

Dr Gaia Nebbia ^12^

**Funding acquisition, Leadership and supervision, Project administration, Samples and logistics, and Sequencing and analysis:**

Ian Johnston ^116^

**Leadership and supervision, Metadata curation, Project administration, Samples and logistics, and Sequencing and analysis:**

Prof Katrina Lythgoe ^5^, Dr M. Estee Torok ^19, 20^ and Prof Ian G Goodfellow ^24^

**Leadership and supervision, Metadata curation, Project administration, Samples and logistics, and Visualisation:**

Dr Jacqui A Prieto ^97, 82^ and Dr Kordo Saeed ^97, 83^

**Leadership and supervision, Metadata curation, Project administration, Sequencing and analysis, and Software and analysis tools:**

Dr David K Jackson ^116^

**Leadership and supervision, Metadata curation, Samples and logistics, Sequencing and analysis, and Visualisation:**

Dr Catherine Houlihan ^96, 94^

**Leadership and supervision, Metadata curation, Sequencing and analysis, Software and analysis tools, and Visualisation:**

Dr Dan Frampton ^94, 95^

**Metadata curation, Project administration, Samples and logistics, Sequencing and analysis, and Software and analysis tools:**

Dr William L Hamilton ^19^ and Dr Adam A Witney ^41^

**Funding acquisition, Samples and logistics, Sequencing and analysis, and Visualisation:**

Dr Giselda Bucca ^101^

**Funding acquisition, Leadership and supervision, Metadata curation, and Project administration:**

Dr Cassie F Pope ^40, 41^

**Funding acquisition, Leadership and supervision, Metadata curation, and Samples and logistics:**

Dr Catherine Moore ^74^

**Funding acquisition, Leadership and supervision, Metadata curation, and Sequencing and analysis:**

Prof Emma C Thomson ^53^

**Funding acquisition, Leadership and supervision, Project administration, and Samples and logistics:**

Dr Ewan M Harrison ^116, 102^

**Funding acquisition, Leadership and supervision, Sequencing and analysis, and Visualisation:**

Prof Colin P Smith ^101^

**Leadership and supervision, Metadata curation, Project administration, and Sequencing and analysis:**

Fiona Rogan ^77^

**Leadership and supervision, Metadata curation, Project administration, and Samples and logistics:**

Shaun M Beckwith ^6^, Abigail Murray ^6^, Dawn Singleton ^6^, Dr Kirstine Eastick ^37^, Dr Liz A Sheridan ^98^, Paul Randell ^99^, Dr Leigh M Jackson ^105^, Dr Cristina V Ariani ^116^ and Dr Sónia Gonçalves ^116^

**Leadership and supervision, Metadata curation, Samples and logistics, and Sequencing and analysis:**

Dr Derek J Fairley ^3, 77^, Prof Matthew W Loose ^18^ and Joanne Watkins ^74^

**Leadership and supervision, Metadata curation, Samples and logistics, and Visualisation:**

Dr Samuel Moses ^25, 106^

**Leadership and supervision, Metadata curation, Sequencing and analysis, and Software and analysis tools:**

Dr Sam Nicholls ^43^, Dr Matthew Bull ^74^ and Dr Roberto Amato ^116^

**Leadership and supervision, Project administration, Samples and logistics, and Sequencing and analysis:**

Prof Darren L Smith ^36, 65, 66^

**Leadership and supervision, Sequencing and analysis, Software and analysis tools, and Visualisation:**

Prof David M Aanensen ^14, 116^ and Dr Jeffrey C Barrett ^116^

**Metadata curation, Project administration, Samples and logistics, and Sequencing and analysis:**

Dr Dinesh Aggarwal ^20, 116, 70^, Dr James G Shepherd ^53^, Dr Martin D Curran ^71^ and Dr Surendra Parmar ^71^

**Metadata curation, Project administration, Sequencing and analysis, and Software and analysis tools:**

Dr Matthew D Parker ^109^

**Metadata curation, Samples and logistics, Sequencing and analysis, and Software and analysis tools:**

Dr Catryn Williams ^74^

**Metadata curation, Samples and logistics, Sequencing and analysis, and Visualisation:**

Dr Sharon Glaysher ^68^

**Metadata curation, Sequencing and analysis, Software and analysis tools, and Visualisation:**

Dr Anthony P Underwood ^14, 116^, Dr Matthew Bashton ^36, 65^, Dr Nicole Pacchiarini ^74^, Dr Katie F Loveson ^84^ and Matthew Byott ^95, 96^

**Project administration, Sequencing and analysis, Software and analysis tools, and Visualisation:**

Dr Alessandro M Carabelli ^20^

**Funding acquisition, Leadership and supervision, and Metadata curation:**

Dr Kate E Templeton ^56, 104^

**Funding acquisition, Leadership and supervision, and Project administration:**

Dr Thushan I de Silva ^109^, Dr Dennis Wang ^109^, Dr Cordelia F Langford ^116^ and John Sillitoe ^116^

**Funding acquisition, Leadership and supervision, and Samples and logistics:**

Prof Rory N Gunson ^55^

**Funding acquisition, Leadership and supervision, and Sequencing and analysis:**

Dr Simon Cottrell ^74^, Dr Justin O’Grady ^75, 103^ and Prof Dominic Kwiatkowski ^116, 108^

**Leadership and supervision, Metadata curation, and Project administration:**

Dr Patrick J Lillie ^37^

**Leadership and supervision, Metadata curation, and Samples and logistics:**

Dr Nicholas Cortes ^33^, Dr Nathan Moore ^33^, Dr Claire Thomas ^33^, Phillipa J Burns ^37^, Dr Tabitha W Mahungu ^80^ and Steven Liggett ^86^

**Leadership and supervision, Metadata curation, and Sequencing and analysis:**

Angela H Beckett ^13, 81^ and Prof Matthew TG Holden ^73^

**Leadership and supervision, Project administration, and Samples and logistics:**

Dr Lisa J Levett ^34^, Dr Husam Osman ^70, 35^ and Dr Mohammed O Hassan-Ibrahim ^99^

**Leadership and supervision, Project administration, and Sequencing and analysis:**

Dr David A Simpson ^77^

**Leadership and supervision, Samples and logistics, and Sequencing and analysis:**

Dr Meera Chand ^72^, Prof Ravi K Gupta ^102^, Prof Alistair C Darby ^107^ and Prof Steve Paterson ^107^

**Leadership and supervision, Sequencing and analysis, and Software and analysis tools:**

Prof Oliver G Pybus ^23^, Dr Erik M Volz ^39^, Prof Daniela de Angelis ^52^, Prof David L Robertson ^53^, Dr Andrew J Page ^75^ and Dr Inigo Martincorena ^116^

**Leadership and supervision, Sequencing and analysis, and Visualisation:**

Dr Louise Aigrain ^116^ and Dr Andrew R Bassett ^116^

**Metadata curation, Project administration, and Samples and logistics:**

Dr Nick Wong ^50^, Dr Yusri Taha ^89^, Michelle J Erkiert ^99^ and Dr Michael H Spencer Chapman ^116, 102^

**Metadata curation, Project administration, and Sequencing and analysis:**

Dr Rebecca Dewar ^56^ and Martin P McHugh ^56, 111^

**Metadata curation, Project administration, and Software and analysis tools:**

Siddharth Mookerjee ^38, 57^

**Metadata curation, Project administration, and Visualisation:**

Stephen Aplin ^97^, Matthew Harvey ^97^, Thea Sass ^97^, Dr Helen Umpleby ^97^ and Helen Wheeler ^97^

**Metadata curation, Samples and logistics, and Sequencing and analysis:**

Dr James P McKenna ^3^, Dr Ben Warne ^9^, Joshua F Taylor ^22^, Yasmin Chaudhry ^24^, Rhys Izuagbe ^24^, Dr Aminu S Jahun ^24^, Dr Gregory R Young ^36, 65^, Dr Claire McMurray ^43^, Dr Clare M McCann ^65, 66^, Dr Andrew Nelson ^65, 66^ and Scott Elliott ^68^

**Metadata curation, Samples and logistics, and Visualisation:**

Hannah Lowe ^25^

**Metadata curation, Sequencing and analysis, and Software and analysis tools:**

Dr Anna Price ^11^, Matthew R Crown ^65^, Dr Sara Rey ^74^, Dr Sunando Roy ^96^ and Dr Ben Temperton ^105^

**Metadata curation, Sequencing and analysis, and Visualisation:**

Dr Sharif Shaaban ^73^ and Dr Andrew R Hesketh ^101^

**Project administration, Samples and logistics, and Sequencing and analysis:**

Dr Kenneth G Laing ^41^, Dr Irene M Monahan ^41^ and Dr Judith Heaney ^95, 96, 34^

**Project administration, Samples and logistics, and Visualisation:**

Dr Emanuela Pelosi ^97^, Siona Silviera ^97^ and Dr Eleri Wilson-Davies ^97^

**Samples and logistics, Software and analysis tools, and Visualisation:**

Dr Helen Fryer ^5^

**Sequencing and analysis, Software and analysis tools, and Visualization:**

Dr Helen Adams ^4^, Dr Louis du Plessis ^23^, Dr Rob Johnson ^39^, Dr William T Harvey ^53, 42^, Dr Joseph Hughes ^53^, Dr Richard J Orton ^53^, Dr Lewis G Spurgin ^59^, Dr Yann Bourgeois ^81^, Dr Chris Ruis ^102^, Áine O'Toole ^104^, Marina Gourtovaia ^116^ and Dr Theo Sanderson ^116^

**Funding acquisition, and Leadership and supervision:**

Dr Christophe Fraser ^5^, Dr Jonathan Edgeworth ^12^, Prof Judith Breuer ^96, 29^, Dr Stephen L Michell ^105^ and Prof John A Todd ^115^

**Funding acquisition, and Project administration:**

Michaela John ^10^ and Dr David Buck ^115^

**Leadership and supervision, and Metadata curation:**

Dr Kavitha Gajee ^37^ and Dr Gemma L Kay ^75^

**Leadership and supervision, and Project administration:**

Prof Sharon J Peacock ^20, 70^ and David Heyburn ^74^

**Leadership and supervision, and Samples and logistics:**

Katie Kitchman ^37^, Prof Alan McNally ^43, 93^, David T Pritchard ^50^, Dr Samir Dervisevic ^58^, Dr Peter Muir ^70^, Dr Esther Robinson ^70, 35^, Dr Barry B Vipond ^70^, Newara A Ramadan ^78^, Dr Christopher Jeanes ^90^, Danni Weldon ^116^, Jana Catalan ^118^ and Neil Jones ^118^

**Leadership and supervision, and Sequencing and analysis:**

Dr Ana da Silva Filipe ^53^, Dr Chris Williams ^74^, Marc Fuchs ^77^, Dr Julia Miskelly ^77^, Dr Aaron R Jeffries ^105^, Karen Oliver ^116^ and Dr Naomi R Park ^116^

**Metadata curation, and Samples and logistics:**

Amy Ash ^1^, Cherian Koshy ^1^, Magdalena Barrow ^7^, Dr Sarah L Buchan ^7^, Dr Anna Mantzouratou ^7^, Dr Gemma Clark ^15^, Dr Christopher W Holmes ^16^, Sharon Campbell ^17^, Thomas Davis ^21^, Ngee Keong Tan ^22^, Dr Julianne R Brown ^29^, Dr Kathryn A Harris ^29, 2^, Stephen P Kidd ^33^, Dr Paul R Grant ^34^, Dr Li Xu-McCrae ^35^, Dr Alison Cox ^38, 63^, Pinglawathee Madona ^38, 63^, Dr Marcus Pond ^38, 63^, Dr Paul A Randell ^38, 63^, Karen T Withell ^48^, Cheryl Williams ^51^, Dr Clive Graham ^60^, Rebecca Denton-Smith ^62^, Emma Swindells ^62^, Robyn Turnbull ^62^, Dr Tim J Sloan ^67^, Dr Andrew Bosworth ^70, 35^, Stephanie Hutchings ^70^, Hannah M Pymont ^70^, Dr Anna Casey ^76^, Dr Liz Ratcliffe ^76^, Dr Christopher R Jones ^79, 105^, Dr Bridget A Knight ^79, 105^, Dr Tanzina Haque ^80^, Dr Jennifer Hart ^80^, Dr Dianne Irish-Tavares ^80^, Eric Witele ^80^, Craig Mower ^86^, Louisa K Watson ^86^, Jennifer Collins ^89^, Gary Eltringham ^89^, Dorian Crudgington ^98^, Ben Macklin ^98^, Prof Miren Iturriza-Gomara ^107^, Dr Anita O Lucaci ^107^ and Dr Patrick C McClure ^113^

**Metadata curation, and Sequencing and analysis:**

Matthew Carlile ^18^, Dr Nadine Holmes ^18^, Dr Christopher Moore ^18^, Dr Nathaniel Storey ^29^, Dr Stefan Rooke ^73^, Dr Gonzalo Yebra ^73^, Dr Noel Craine ^74^, Malorie Perry ^74^, Dr Nabil-Fareed Alikhan ^75^, Dr Stephen Bridgett ^77^, Kate F Cook ^84^, Christopher Fearn ^84^, Dr Salman Goudarzi ^84^, Prof Ronan A Lyons ^88^, Dr Thomas Williams ^104^, Dr Sam T Haldenby ^107^, Jillian Durham ^116^ and Dr Steven Leonard ^116^

**Metadata curation, and Software and analysis tools:**

Robert M Davies ^116^

**Project administration, and Samples and logistics:**

Dr Rahul Batra ^12^, Beth Blane ^20^, Dr Moira J Spyer ^30, 95, 96^, Perminder Smith ^32, 112^, Mehmet Yavus ^85, 109^, Dr Rachel J Williams ^96^, Dr Adhyana IK Mahanama ^97^, Dr Buddhini Samaraweera ^97^, Sophia T Girgis ^102^, Samantha E Hansford ^109^, Dr Angie Green ^115^, Dr Charlotte Beaver ^116^, Katherine L Bellis ^116, 102^, Matthew J Dorman ^116^, Sally Kay ^116^, Liam Prestwood ^116^ and Dr Shavanthi Rajatileka ^116^

**Project administration, and Sequencing and analysis:**

Dr Joshua Quick ^43^

**Project administration, and Software and analysis tools:**

Radoslaw Poplawski ^43^

**Samples and logistics, and Sequencing and analysis:**

Dr Nicola Reynolds ^8^, Andrew Mack ^11^, Dr Arthur Morriss ^11^, Thomas Whalley ^11^, Bindi Patel ^12^, Dr Iliana Georgana ^24^, Dr Myra Hosmillo ^24^, Malte L Pinckert ^24^, Dr Joanne Stockton ^43^, Dr John H Henderson ^65^, Amy Hollis ^65^, Dr William Stanley ^65^, Dr Wen C Yew ^65^, Dr Richard Myers ^72^, Dr Alicia Thornton ^72^, Alexander Adams ^74^, Tara Annett ^74^, Dr Hibo Asad ^74^, Alec Birchley ^74^, Jason Coombes ^74^, Johnathan M Evans ^74^, Laia Fina ^74^, Bree Gatica-Wilcox ^74^, Lauren Gilbert ^74^, Lee Graham ^74^, Jessica Hey ^74^, Ember Hilvers ^74^, Sophie Jones ^74^, Hannah Jones ^74^, Sara Kumziene-Summerhayes ^74^, Dr Caoimhe McKerr ^74^, Jessica Powell ^74^, Georgia Pugh ^74^, Sarah Taylor ^74^, Alexander J Trotter ^75^, Charlotte A Williams ^96^, Leanne M Kermack ^102^, Benjamin H Foulkes ^109^, Marta Gallis ^109^, Hailey R Hornsby ^109^, Stavroula F Louka ^109^, Dr Manoj Pohare ^109^, Paige Wolverson ^109^, Peijun Zhang ^109^, George MacIntyre-Cockett ^115^, Amy Trebes ^115^, Dr Robin J Moll ^116^, Lynne Ferguson ^117^, Dr Emily J Goldstein ^117^, Dr Alasdair Maclean ^117^ and Dr Rachael Tomb ^117^

**Samples and logistics, and Software and analysis tools:**

Dr Igor Starinskij ^53^

**Sequencing and analysis, and Software and analysis tools:**

Laura Thomson ^5^, Joel Southgate ^11, 74^, Dr Moritz UG Kraemer ^23^, Dr Jayna Raghwani ^23^, Dr Alex E Zarebski ^23^, Olivia Boyd ^39^, Lily Geidelberg ^39^, Dr Chris J Illingworth ^52^, Dr Chris Jackson ^52^, Dr David Pascall ^52^, Dr Sreenu Vattipally ^53^, Timothy M Freeman ^109^, Dr Sharon N Hsu ^109^, Dr Benjamin B Lindsey ^109^, Dr Keith James ^116^, Kevin Lewis ^116^, Gerry Tonkin-Hill ^116^ and Dr Jaime M Tovar-Corona ^116^

**Sequencing and analysis, and Visualisation:**

MacGregor Cox ^20^

**Software and analysis tools, and Visualisation:**

Dr Khalil Abudahab ^14, 116^, Mirko Menegazzo ^14^, Ben EW Taylor MEng ^14, 116^, Dr Corin A Yeats ^14^, Afrida Mukaddas ^53^, Derek W Wright ^53^, Dr Leonardo de Oliveira Martins ^75^, Dr Rachel Colquhoun ^104^, Verity Hill ^104^, Dr Ben Jackson ^104^, Dr JT McCrone ^104^, Dr Nathan Medd ^104^, Dr Emily Scher ^104^ and Jon-Paul Keatley ^116^

**Leadership and supervision:**

Dr Tanya Curran ^3^, Dr Sian Morgan ^10^, Prof Patrick Maxwell ^20^, Prof Ken Smith ^20^, Dr Sahar Eldirdiri ^21^, Anita Kenyon ^21^, Prof Alison H Holmes ^38, 57^, Dr James R Price ^38, 57^, Dr Tim Wyatt ^69^, Dr Alison E Mather ^75^, Dr Timofey Skvortsov ^77^ and Prof John A Hartley ^96^

**Metadata curation:**

Prof Martyn Guest ^11^, Dr Christine Kitchen ^11^, Dr Ian Merrick ^11^, Robert Munn ^11^, Dr Beatrice Bertolusso ^33^, Dr Jessica Lynch ^33^, Dr Gabrielle Vernet ^33^, Stuart Kirk ^34^, Dr Elizabeth Wastnedge ^56^, Dr Rachael Stanley ^58^, Giles Idle ^64^, Dr Declan T Bradley ^69, 77^, Dr Jennifer Poyner ^79^ and Matilde Mori ^110^

**Project administration:**

Owen Jones ^11^, Victoria Wright ^18^, Ellena Brooks ^20^, Carol M Churcher ^20^, Mireille Fragakis ^20^, Dr Katerina Galai ^20, 70^, Dr Andrew Jermy ^20^, Sarah Judges ^20^, Georgina M McManus ^20^, Kim S Smith ^20^, Dr Elaine Westwick ^20^, Dr Stephen W Attwood ^23^, Dr Frances Bolt ^38, 57^, Dr Alisha Davies ^74^, Elen De Lacy ^74^, Fatima Downing ^74^, Sue Edwards ^74^, Lizzie Meadows ^75^, Sarah Jeremiah ^97^, Dr Nikki Smith ^109^ and Luke Foulser ^116^

**Samples and logistics:**

Dr Themoula Charalampous ^12, 46^, Amita Patel ^12^, Dr Louise Berry ^15^, Dr Tim Boswell ^15^, Dr Vicki M Fleming ^15^, Dr Hannah C Howson-Wells ^15^, Dr Amelia Joseph ^15^, Manjinder Khakh ^15^, Dr Michelle M Lister ^15^, Paul W Bird ^16^, Karlie Fallon ^16^, Thomas Helmer ^16^, Dr Claire L McMurray ^16^, Mina Odedra ^16^, Jessica Shaw ^16^, Dr Julian W Tang ^16^, Nicholas J Willford ^16^, Victoria Blakey ^17^, Dr Veena Raviprakash ^17^, Nicola Sheriff ^17^, Lesley-Anne Williams ^17^, Theresa Feltwell ^20^, Dr Luke Bedford ^26^, Dr James S Cargill ^27^, Warwick Hughes ^27^, Dr Jonathan Moore ^28^, Susanne Stonehouse ^28^, Laura Atkinson ^29^, Jack CD Lee ^29^, Dr Divya Shah ^29^, Adela Alcolea-Medina ^32, 112^, Natasha Ohemeng-Kumi ^32, 112^, John Ramble ^32, 112^, Jasveen Sehmi ^32, 112^, Dr Rebecca Williams ^33^, Wendy Chatterton ^34^, Monika Pusok ^34^, William Everson ^37^, Anibolina Castigador ^44^, Emily Macnaughton ^44^, Dr Kate El Bouzidi ^45^, Dr Temi Lampejo ^45^, Dr Malur Sudhanva ^45^, Cassie Breen ^47^, Dr Graciela Sluga ^48^, Dr Shazaad SY Ahmad ^49, 70^, Dr Ryan P George ^49^, Dr Nicholas W Machin ^49, 70^, Debbie Binns ^50^, Victoria James ^50^, Dr Rachel Blacow ^55^, Dr Lindsay Coupland ^58^, Dr Louise Smith ^59^, Dr Edward Barton ^60^, Debra Padgett ^60^, Garren Scott ^60^, Dr Aidan Cross ^61^, Dr Mariyam Mirfenderesky ^61^, Jane Greenaway ^62^, Kevin Cole ^64^, Phillip Clarke ^67^, Nichola Duckworth ^67^, Sarah Walsh ^67^, Kelly Bicknell ^68^, Robert Impey ^68^, Dr Sarah Wyllie ^68^, Richard Hopes ^70^, Dr Chloe Bishop ^72^, Dr Vicki Chalker ^72^, Dr Ian Harrison ^72^, Laura Gifford ^74^, Dr Zoltan Molnar ^77^, Dr Cressida Auckland ^79^, Dr Cariad Evans ^85, 109^, Dr Kate Johnson ^85, 109^, Dr David G Partridge ^85, 109^, Dr Mohammad Raza ^85, 109^, Paul Baker ^86^, Prof Stephen Bonner ^86^, Sarah Essex ^86^, Leanne J Murray ^86^, Andrew I Lawton ^87^, Dr Shirelle Burton-Fanning ^89^, Dr Brendan AI Payne ^89^, Dr Sheila Waugh ^89^, Andrea N Gomes ^91^, Maimuna Kimuli ^91^, Darren R Murray ^91^, Paula Ashfield ^92^, Dr Donald Dobie ^92^, Dr Fiona Ashford ^93^, Dr Angus Best ^93^, Dr Liam Crawford ^93^, Dr Nicola Cumley ^93^, Dr Megan Mayhew ^93^, Dr Oliver Megram ^93^, Dr Jeremy Mirza ^93^, Dr Emma Moles-Garcia ^93^, Dr Benita Percival ^93^, Megan Driscoll ^96^, Leah Ensell ^96^, Dr Helen L Lowe ^96^, Laurentiu Maftei ^96^, Matteo Mondani ^96^, Nicola J Chaloner ^99^, Benjamin J Cogger ^99^, Lisa J Easton ^99^, Hannah Huckson ^99^, Jonathan Lewis ^99^, Sarah Lowdon ^99^, Cassandra S Malone ^99^, Florence Munemo ^99^, Manasa Mutingwende ^99^, Roberto Nicodemi ^99^, Olga Podplomyk ^99^, Thomas Somassa ^99^, Dr Andrew Beggs ^100^, Dr Alex Richter ^100^, Claire Cormie ^102^, Joana Dias ^102^, Sally Forrest ^102^, Dr Ellen E Higginson ^102^, Mailis Maes ^102^, Jamie Young ^102^, Dr Rose K Davidson ^103^, Kathryn A Jackson ^107^, Dr Lance Turtle ^107^, Dr Alexander J Keeley ^109^, Prof Jonathan Ball ^113^, Timothy Byaruhanga ^113^, Dr Joseph G Chappell ^113^, Jayasree Dey ^113^, Jack D Hill ^113^, Emily J Park ^113^, Arezou Fanaie ^114^, Rachel A Hilson ^114^, Geraldine Yaze ^114^ and Stephanie Lo ^116^

**Sequencing and analysis:**

Safiah Afifi ^10^, Robert Beer ^10^, Joshua Maksimovic ^10^, Kathryn McCluggage ^10^, Karla Spellman ^10^, Catherine Bresner ^11^, William Fuller ^11^, Dr Angela Marchbank ^11^, Trudy Workman ^11^, Dr Ekaterina Shelest ^13, 81^, Dr Johnny Debebe ^18^, Dr Fei Sang ^18^, Dr Marina Escalera Zamudio ^23^, Dr Sarah Francois ^23^, Bernardo Gutierrez ^23^, Dr Tetyana I Vasylyeva ^23^, Dr Flavia Flaviani ^31^, Dr Manon Ragonnet-Cronin ^39^, Dr Katherine L Smollett ^42^, Alice Broos ^53^, Daniel Mair ^53^, Jenna Nichols ^53^, Dr Kyriaki Nomikou ^53^, Dr Lily Tong ^53^, Ioulia Tsatsani ^53^, Prof Sarah O'Brien ^54^, Prof Steven Rushton ^54^, Dr Roy Sanderson ^54^, Dr Jon Perkins ^55^, Seb Cotton ^56^, Abbie Gallagher ^56^, Dr Elias Allara ^70, 102^, Clare Pearson ^70, 102^, Dr David Bibby ^72^, Dr Gavin Dabrera ^72^, Dr Nicholas Ellaby ^72^, Dr Eileen Gallagher ^72^, Dr Jonathan Hubb ^72^, Dr Angie Lackenby ^72^, Dr David Lee ^72^, Nikos Manesis ^72^, Dr Tamyo Mbisa ^72^, Dr Steven Platt ^72^, Katherine A Twohig ^72^, Dr Mari Morgan ^74^, Alp Aydin ^75^, David J Baker ^75^, Dr Ebenezer Foster-Nyarko ^75^, Dr Sophie J Prosolek ^75^, Steven Rudder ^75^, Chris Baxter ^77^, Sílvia F Carvalho ^77^, Dr Deborah Lavin ^77^, Dr Arun Mariappan ^77^, Dr Clara Radulescu ^77^, Dr Aditi Singh ^77^, Miao Tang ^77^, Helen Morcrette ^79^, Nadua Bayzid ^96^, Marius Cotic ^96^, Dr Carlos E Balcazar ^104^, Dr Michael D Gallagher ^104^, Dr Daniel Maloney ^104^, Thomas D Stanton ^104^, Dr Kathleen A Williamson ^104^, Dr Robin Manley ^105^, Michelle L Michelsen ^105^, Dr Christine M Sambles ^105^, Dr David J Studholme ^105^, Joanna Warwick-Dugdale ^105^, Richard Eccles ^107^, Matthew Gemmell ^107^, Dr Richard Gregory ^107^, Dr Margaret Hughes ^107^, Charlotte Nelson ^107^, Dr Lucille Rainbow ^107^, Dr Edith E Vamos ^107^, Hermione J Webster ^107^, Dr Mark Whitehead ^107^, Claudia Wierzbicki ^107^, Dr Adrienn Angyal ^109^, Dr Luke R Green ^109^, Dr Max Whiteley ^109^, Emma Betteridge ^116^, Dr Iraad F Bronner ^116^, Ben W Farr ^116^, Scott Goodwin ^116^, Dr Stefanie V Lensing ^116^, Shane A McCarthy ^116, 102^, Dr Michael A Quail ^116^, Diana Rajan ^116^, Dr Nicholas M Redshaw ^116^, Carol Scott ^116^, Lesley Shirley ^116^ and Scott AJ Thurston ^116^

**Software and analysis tools:**

Dr Will Rowe ^43^, Amy Gaskin ^74^, Dr Thanh Le-Viet ^75^, James Bonfield ^116^, Jennifier Liddle ^116^ and Andrew Whitwham ^116^

**1** Barking, Havering and Redbridge University Hospitals NHS Trust, **2** Barts Health NHS Trust, **3** Belfast Health & Social Care Trust, **4** Betsi Cadwaladr University Health Board, **5** Big Data Institute, Nuffield Department of Medicine, University of Oxford, **6** Blackpool Teaching Hospitals NHS Foundation Trust, **7** Bournemouth University, **8** Cambridge Stem Cell Institute, University of Cambridge, **9** Cambridge University Hospitals NHS Foundation Trust, **10** Cardiff and Vale University Health Board, **11** Cardiff University, **12** Centre for Clinical Infection and Diagnostics Research, Department of Infectious Diseases, Guy's and St Thomas' NHS Foundation Trust, **13** Centre for Enzyme Innovation, University of Portsmouth, **14** Centre for Genomic Pathogen Surveillance, University of Oxford, **15** Clinical Microbiology Department, Queens Medical Centre, Nottingham University Hospitals NHS Trust, **16** Clinical Microbiology, University Hospitals of Leicester NHS Trust, **17** County Durham and Darlington NHS Foundation Trust, **18** Deep Seq, School of Life Sciences, Queens Medical Centre, University of Nottingham, **19** Department of Infectious Diseases and Microbiology, Cambridge University Hospitals NHS Foundation Trust, **20** Department of Medicine, University of Cambridge, **21** Department of Microbiology, Kettering General Hospital, **22** Department of Microbiology, South West London Pathology, **23** Department of Zoology, University of Oxford, **24** Division of Virology, Department of Pathology, University of Cambridge, **25** East Kent Hospitals University NHS Foundation Trust, **26** East Suffolk and North Essex NHS Foundation Trust, **27** East Sussex Healthcare NHS Trust, **28** Gateshead Health NHS Foundation Trust, **29** Great Ormond Street Hospital for Children NHS Foundation Trust, **30** Great Ormond Street Institute of Child Health (GOS ICH), University College London (UCL), **31** Guy's and St. Thomas’ Biomedical Research Centre, **32** Guy's and St. Thomas’ NHS Foundation Trust, **33** Hampshire Hospitals NHS Foundation Trust, **34** Health Services Laboratories, **35** Heartlands Hospital, Birmingham, **36** Hub for Biotechnology in the Built Environment, Northumbria University, **37** Hull University Teaching Hospitals NHS Trust, **38** Imperial College Healthcare NHS Trust, **39** Imperial College London, **40** Infection Care Group, St George’s University Hospitals NHS Foundation Trust, **41** Institute for Infection and Immunity, St George’s University of London, **42** Institute of Biodiversity, Animal Health & Comparative Medicine, **43** Institute of Microbiology and Infection, University of Birmingham, **44** Isle of Wight NHS Trust, **45** King's College Hospital NHS Foundation Trust, **46** King's College London, **47** Liverpool Clinical Laboratories, **48** Maidstone and Tunbridge Wells NHS Trust, **49** Manchester University NHS Foundation Trust, **50** Microbiology Department, Buckinghamshire Healthcare NHS Trust, **51** Microbiology, Royal Oldham Hospital, **52** MRC Biostatistics Unit, University of Cambridge, **53** MRC-University of Glasgow Centre for Virus Research, **54** Newcastle University, **55** NHS Greater Glasgow and Clyde, **56** NHS Lothian, **57** NIHR Health Protection Research Unit in HCAI and AMR, Imperial College London, **58** Norfolk and Norwich University Hospitals NHS Foundation Trust, **59** Norfolk County Council, **60** North Cumbria Integrated Care NHS Foundation Trust, **61** North Middlesex University Hospital NHS Trust, **62** North Tees and Hartlepool NHS Foundation Trust, **63** North West London Pathology, **64** Northumbria Healthcare NHS Foundation Trust, **65** Northumbria University, **66** NU-OMICS, Northumbria University, **67** Path Links, Northern Lincolnshire and Goole NHS Foundation Trust, **68** Portsmouth Hospitals University NHS Trust, **69** Public Health Agency, Northern Ireland, **70** Public Health England, **71** Public Health England, Cambridge, **72** Public Health England, Colindale, **73** Public Health Scotland, **74** Public Health Wales, **75** Quadram Institute Bioscience, **76** Queen Elizabeth Hospital, Birmingham, **77** Queen's University Belfast, **78** Royal Brompton and Harefield Hospitals, **79** Royal Devon and Exeter NHS Foundation Trust, **80** Royal Free London NHS Foundation Trust, **81** School of Biological Sciences, University of Portsmouth, **82** School of Health Sciences, University of Southampton, **83** School of Medicine, University of Southampton, **84** School of Pharmacy & Biomedical Sciences, University of Portsmouth, **85** Sheffield Teaching Hospitals NHS Foundation Trust, **86** South Tees Hospitals NHS Foundation Trust, **87** Southwest Pathology Services, **88** Swansea University, **89** The Newcastle upon Tyne Hospitals NHS Foundation Trust, **90** The Queen Elizabeth Hospital King's Lynn NHS Foundation Trust, **91** The Royal Marsden NHS Foundation Trust, **92** The Royal Wolverhampton NHS Trust, **93** Turnkey Laboratory, University of Birmingham, **94** University College London Division of Infection and Immunity**, 95** University College London Hospital Advanced Pathogen Diagnostics Unit**, 96** University College London Hospitals NHS Foundation Trust, **97** University Hospital Southampton NHS Foundation Trust, **98** University Hospitals Dorset NHS Foundation Trust, **99** University Hospitals Sussex NHS Foundation Trust, **100** University of Birmingham, **101** University of Brighton, **102** University of Cambridge, **103** University of East Anglia, **104** University of Edinburgh, **105** University of Exeter, **106** University of Kent, **107** University of Liverpool, **108** University of Oxford, **109** University of Sheffield, **110** University of Southampton, **111** University of St Andrews, **112** Viapath, Guy's and St Thomas' NHS Foundation Trust, and King's College Hospital NHS Foundation Trust, **113** Virology, School of Life Sciences, Queens Medical Centre, University of Nottingham, **114** Watford General Hospital, **115** Wellcome Centre for Human Genetics, Nuffield Department of Medicine, University of Oxford, **116** Wellcome Sanger Institute, **117** West of Scotland Specialist Virology Centre, NHS Greater Glasgow and Clyde, **118** Whittington Health NHS Trust
