# Supplementary material for: Tracking SARS-CoV-2 mutations and variants through the COG-UK-Mutation Explorer
Source: Virus Evol. 2022 Mar 18;8(1):veac023. doi: 10.1093/ve/veac023 (PMC9037374; doi:10.1093/ve/veac023)
Supplement: veac023_Supp [file veac023_supp.zip › Appendix 1.docx]

**The COVID-19 Genomics UK (COG-UK) consortium**

**June 2021 V.1**

**Funding acquisition, Leadership and supervision, Metadata curation, Project administration, Samples and logistics, Sequencing and analysis, Software and analysis tools, and Visualisation:**

Dr Samuel C Robson ^13, 84^

**Funding acquisition, Leadership and supervision, Metadata curation, Project administration, Samples and logistics, Sequencing and analysis, and Software and analysis tools:**

Dr Thomas R Connor ^11, 74^ and Prof Nicholas J Loman ^43^

**Leadership and supervision, Metadata curation, Project administration, Samples and logistics, Sequencing and analysis, Software and analysis tools, and Visualisation:**

Dr Tanya Golubchik ^5^

**Funding acquisition, Leadership and supervision, Metadata curation, Samples and logistics, Sequencing and analysis, and Visualisation:**

Dr Rocio T Martinez Nunez ^46^

**Funding acquisition, Leadership and supervision, Project administration, Samples and logistics, Sequencing and analysis, and Software and analysis tools:**

Dr David Bonsall ^5^

**Funding acquisition, Leadership and supervision, Project administration, Sequencing and analysis, Software and analysis tools, and Visualisation:**

Prof Andrew Rambaut ^104^

**Funding acquisition, Metadata curation, Project administration, Samples and logistics, Sequencing and analysis, and Software and analysis tools:**

Dr Luke B Snell ^12^

**Leadership and supervision, Metadata curation, Project administration, Samples and logistics, Software and analysis tools, and Visualisation:**

Rich Livett ^116^

**Funding acquisition, Leadership and supervision, Metadata curation, Project administration, and Samples and logistics:**

Dr Catherine Ludden ^20, 70^

**Funding acquisition, Leadership and supervision, Metadata curation, Samples and logistics, and Sequencing and analysis:**

Dr Sally Corden ^74^ and Dr Eleni Nastouli ^96, 95, 30^

**Funding acquisition, Leadership and supervision, Metadata curation, Sequencing and analysis, and Software and analysis tools:**

Dr Gaia Nebbia ^12^

**Funding acquisition, Leadership and supervision, Project administration, Samples and logistics, and Sequencing and analysis:**

Ian Johnston ^116^

**Leadership and supervision, Metadata curation, Project administration, Samples and logistics, and Sequencing and analysis:**

Prof Katrina Lythgoe ^5^, Dr M. Estee Torok ^19, 20^ and Prof Ian G Goodfellow ^24^

**Leadership and supervision, Metadata curation, Project administration, Samples and logistics, and Visualisation:**

Dr Jacqui A Prieto ^97, 82^ and Dr Kordo Saeed ^97, 83^

**Leadership and supervision, Metadata curation, Project administration, Sequencing and analysis, and Software and analysis tools:**

Dr David K Jackson ^116^

**Leadership and supervision, Metadata curation, Samples and logistics, Sequencing and analysis, and Visualisation:**

Dr Catherine Houlihan ^96, 94^

**Leadership and supervision, Metadata curation, Sequencing and analysis, Software and analysis tools, and Visualisation:**

Dr Dan Frampton ^94, 95^

**Metadata curation, Project administration, Samples and logistics, Sequencing and analysis, and Software and analysis tools:**

Dr William L Hamilton ^19^ and Dr Adam A Witney ^41^

**Funding acquisition, Samples and logistics, Sequencing and analysis, and Visualisation:**

Dr Giselda Bucca ^101^

**Funding acquisition, Leadership and supervision, Metadata curation, and Project administration:**

Dr Cassie F Pope ^40, 41^

**Funding acquisition, Leadership and supervision, Metadata curation, and Samples and logistics:**

Dr Catherine Moore ^74^

**Funding acquisition, Leadership and supervision, Metadata curation, and Sequencing and analysis:**

Prof Emma C Thomson ^53^

**Funding acquisition, Leadership and supervision, Project administration, and Samples and logistics:**

Dr Ewan M Harrison ^116, 102^

**Funding acquisition, Leadership and supervision, Sequencing and analysis, and Visualisation:**

Prof Colin P Smith ^101^

**Leadership and supervision, Metadata curation, Project administration, and Sequencing and analysis:**

Fiona Rogan ^77^

**Leadership and supervision, Metadata curation, Project administration, and Samples and logistics:**

Shaun M Beckwith ^6^, Abigail Murray ^6^, Dawn Singleton ^6^, Dr Kirstine Eastick ^37^, Dr Liz A Sheridan ^98^, Paul Randell ^99^, Dr Leigh M Jackson ^105^, Dr Cristina V Ariani ^116^ and Dr Sónia Gonçalves ^116^

**Leadership and supervision, Metadata curation, Samples and logistics, and Sequencing and analysis:**

Dr Derek J Fairley ^3, 77^, Prof Matthew W Loose ^18^ and Joanne Watkins ^74^

**Leadership and supervision, Metadata curation, Samples and logistics, and Visualisation:**

Dr Samuel Moses ^25, 106^

**Leadership and supervision, Metadata curation, Sequencing and analysis, and Software and analysis tools:**

Dr Sam Nicholls ^43^, Dr Matthew Bull ^74^ and Dr Roberto Amato ^116^

**Leadership and supervision, Project administration, Samples and logistics, and Sequencing and analysis:**

Prof Darren L Smith ^36, 65, 66^

**Leadership and supervision, Sequencing and analysis, Software and analysis tools, and Visualisation:**

Prof David M Aanensen ^14, 116^ and Dr Jeffrey C Barrett ^116^

**Metadata curation, Project administration, Samples and logistics, and Sequencing and analysis:**

Dr Dinesh Aggarwal ^20, 116, 70^, Dr James G Shepherd ^53^, Dr Martin D Curran ^71^ and Dr Surendra Parmar ^71^

**Metadata curation, Project administration, Sequencing and analysis, and Software and analysis tools:**

Dr Matthew D Parker ^109^

**Metadata curation, Samples and logistics, Sequencing and analysis, and Software and analysis tools:**

Dr Catryn Williams ^74^

**Metadata curation, Samples and logistics, Sequencing and analysis, and Visualisation:**

Dr Sharon Glaysher ^68^

**Metadata curation, Sequencing and analysis, Software and analysis tools, and Visualisation:**

Dr Anthony P Underwood ^14, 116^, Dr Matthew Bashton ^36, 65^, Dr Nicole Pacchiarini ^74^, Dr Katie F Loveson ^84^ and Matthew Byott ^95, 96^

**Project administration, Sequencing and analysis, Software and analysis tools, and Visualisation:**

Dr Alessandro M Carabelli ^20^

**Funding acquisition, Leadership and supervision, and Metadata curation:**

Dr Kate E Templeton ^56, 104^

**Funding acquisition, Leadership and supervision, and Project administration:**

Dr Thushan I de Silva ^109^, Dr Dennis Wang ^109^, Dr Cordelia F Langford ^116^ and John Sillitoe ^116^

**Funding acquisition, Leadership and supervision, and Samples and logistics:**

Prof Rory N Gunson ^55^

**Funding acquisition, Leadership and supervision, and Sequencing and analysis:**

Dr Simon Cottrell ^74^, Dr Justin O’Grady ^75, 103^ and Prof Dominic Kwiatkowski ^116, 108^

**Leadership and supervision, Metadata curation, and Project administration:**

Dr Patrick J Lillie ^37^

**Leadership and supervision, Metadata curation, and Samples and logistics:**

Dr Nicholas Cortes ^33^, Dr Nathan Moore ^33^, Dr Claire Thomas ^33^, Phillipa J Burns ^37^, Dr Tabitha W Mahungu ^80^ and Steven Liggett ^86^

**Leadership and supervision, Metadata curation, and Sequencing and analysis:**

Angela H Beckett ^13, 81^ and Prof Matthew TG Holden ^73^

**Leadership and supervision, Project administration, and Samples and logistics:**

Dr Lisa J Levett ^34^, Dr Husam Osman ^70, 35^ and Dr Mohammed O Hassan-Ibrahim ^99^

**Leadership and supervision, Project administration, and Sequencing and analysis:**

Dr David A Simpson ^77^

**Leadership and supervision, Samples and logistics, and Sequencing and analysis:**

Dr Meera Chand ^72^, Prof Ravi K Gupta ^102^, Prof Alistair C Darby ^107^ and Prof Steve Paterson ^107^

**Leadership and supervision, Sequencing and analysis, and Software and analysis tools:**

Prof Oliver G Pybus ^23^, Dr Erik M Volz ^39^, Prof Daniela de Angelis ^52^, Prof David L Robertson ^53^, Dr Andrew J Page ^75^ and Dr Inigo Martincorena ^116^

**Leadership and supervision, Sequencing and analysis, and Visualisation:**

Dr Louise Aigrain ^116^ and Dr Andrew R Bassett ^116^

**Metadata curation, Project administration, and Samples and logistics:**

Dr Nick Wong ^50^, Dr Yusri Taha ^89^, Michelle J Erkiert ^99^ and Dr Michael H Spencer Chapman ^116, 102^

**Metadata curation, Project administration, and Sequencing and analysis:**

Dr Rebecca Dewar ^56^ and Martin P McHugh ^56, 111^

**Metadata curation, Project administration, and Software and analysis tools:**

Siddharth Mookerjee ^38, 57^

**Metadata curation, Project administration, and Visualisation:**

Stephen Aplin ^97^, Matthew Harvey ^97^, Thea Sass ^97^, Dr Helen Umpleby ^97^ and Helen Wheeler ^97^

**Metadata curation, Samples and logistics, and Sequencing and analysis:**

Dr James P McKenna ^3^, Dr Ben Warne ^9^, Joshua F Taylor ^22^, Yasmin Chaudhry ^24^, Rhys Izuagbe ^24^, Dr Aminu S Jahun ^24^, Dr Gregory R Young ^36, 65^, Dr Claire McMurray ^43^, Dr Clare M McCann ^65, 66^, Dr Andrew Nelson ^65, 66^ and Scott Elliott ^68^

**Metadata curation, Samples and logistics, and Visualisation:**

Hannah Lowe ^25^

**Metadata curation, Sequencing and analysis, and Software and analysis tools:**

Dr Anna Price ^11^, Matthew R Crown ^65^, Dr Sara Rey ^74^, Dr Sunando Roy ^96^ and Dr Ben Temperton ^105^

**Metadata curation, Sequencing and analysis, and Visualisation:**

Dr Sharif Shaaban ^73^ and Dr Andrew R Hesketh ^101^

**Project administration, Samples and logistics, and Sequencing and analysis:**

Dr Kenneth G Laing ^41^, Dr Irene M Monahan ^41^ and Dr Judith Heaney ^95, 96, 34^

**Project administration, Samples and logistics, and Visualisation:**

Dr Emanuela Pelosi ^97^, Siona Silviera ^97^ and Dr Eleri Wilson-Davies ^97^

**Samples and logistics, Software and analysis tools, and Visualisation:**

Dr Helen Fryer ^5^

**Sequencing and analysis, Software and analysis tools, and Visualization:**

Dr Helen Adams ^4^, Dr Louis du Plessis ^23^, Dr Rob Johnson ^39^, Dr William T Harvey ^53, 42^, Dr Joseph Hughes ^53^, Dr Richard J Orton ^53^, Dr Lewis G Spurgin ^59^, Dr Yann Bourgeois ^81^, Dr Chris Ruis ^102^, Áine O'Toole ^104^, Marina Gourtovaia ^116^ and Dr Theo Sanderson ^116^

**Funding acquisition, and Leadership and supervision:**

Dr Christophe Fraser ^5^, Dr Jonathan Edgeworth ^12^, Prof Judith Breuer ^96, 29^, Dr Stephen L Michell ^105^ and Prof John A Todd ^115^

**Funding acquisition, and Project administration:**

Michaela John ^10^ and Dr David Buck ^115^

**Leadership and supervision, and Metadata curation:**

Dr Kavitha Gajee ^37^ and Dr Gemma L Kay ^75^

**Leadership and supervision, and Project administration:**

Prof Sharon J Peacock ^20, 70^ and David Heyburn ^74^

**Leadership and supervision, and Samples and logistics:**

Katie Kitchman ^37^, Prof Alan McNally ^43, 93^, David T Pritchard ^50^, Dr Samir Dervisevic ^58^, Dr Peter Muir ^70^, Dr Esther Robinson ^70, 35^, Dr Barry B Vipond ^70^, Newara A Ramadan ^78^, Dr Christopher Jeanes ^90^, Danni Weldon ^116^, Jana Catalan ^118^ and Neil Jones ^118^

**Leadership and supervision, and Sequencing and analysis:**

Dr Ana da Silva Filipe ^53^, Dr Chris Williams ^74^, Marc Fuchs ^77^, Dr Julia Miskelly ^77^, Dr Aaron R Jeffries ^105^, Karen Oliver ^116^ and Dr Naomi R Park ^116^

**Metadata curation, and Samples and logistics:**

Amy Ash ^1^, Cherian Koshy ^1^, Magdalena Barrow ^7^, Dr Sarah L Buchan ^7^, Dr Anna Mantzouratou ^7^, Dr Gemma Clark ^15^, Dr Christopher W Holmes ^16^, Sharon Campbell ^17^, Thomas Davis ^21^, Ngee Keong Tan ^22^, Dr Julianne R Brown ^29^, Dr Kathryn A Harris ^29, 2^, Stephen P Kidd ^33^, Dr Paul R Grant ^34^, Dr Li Xu-McCrae ^35^, Dr Alison Cox ^38, 63^, Pinglawathee Madona ^38, 63^, Dr Marcus Pond ^38, 63^, Dr Paul A Randell ^38, 63^, Karen T Withell ^48^, Cheryl Williams ^51^, Dr Clive Graham ^60^, Rebecca Denton-Smith ^62^, Emma Swindells ^62^, Robyn Turnbull ^62^, Dr Tim J Sloan ^67^, Dr Andrew Bosworth ^70, 35^, Stephanie Hutchings ^70^, Hannah M Pymont ^70^, Dr Anna Casey ^76^, Dr Liz Ratcliffe ^76^, Dr Christopher R Jones ^79, 105^, Dr Bridget A Knight ^79, 105^, Dr Tanzina Haque ^80^, Dr Jennifer Hart ^80^, Dr Dianne Irish-Tavares ^80^, Eric Witele ^80^, Craig Mower ^86^, Louisa K Watson ^86^, Jennifer Collins ^89^, Gary Eltringham ^89^, Dorian Crudgington ^98^, Ben Macklin ^98^, Prof Miren Iturriza-Gomara ^107^, Dr Anita O Lucaci ^107^ and Dr Patrick C McClure ^113^

**Metadata curation, and Sequencing and analysis:**

Matthew Carlile ^18^, Dr Nadine Holmes ^18^, Dr Christopher Moore ^18^, Dr Nathaniel Storey ^29^, Dr Stefan Rooke ^73^, Dr Gonzalo Yebra ^73^, Dr Noel Craine ^74^, Malorie Perry ^74^, Dr Nabil-Fareed Alikhan ^75^, Dr Stephen Bridgett ^77^, Kate F Cook ^84^, Christopher Fearn ^84^, Dr Salman Goudarzi ^84^, Prof Ronan A Lyons ^88^, Dr Thomas Williams ^104^, Dr Sam T Haldenby ^107^, Jillian Durham ^116^ and Dr Steven Leonard ^116^

**Metadata curation, and Software and analysis tools:**

Robert M Davies ^116^

**Project administration, and Samples and logistics:**

Dr Rahul Batra ^12^, Beth Blane ^20^, Dr Moira J Spyer ^30, 95, 96^, Perminder Smith ^32, 112^, Mehmet Yavus ^85, 109^, Dr Rachel J Williams ^96^, Dr Adhyana IK Mahanama ^97^, Dr Buddhini Samaraweera ^97^, Sophia T Girgis ^102^, Samantha E Hansford ^109^, Dr Angie Green ^115^, Dr Charlotte Beaver ^116^, Katherine L Bellis ^116, 102^, Matthew J Dorman ^116^, Sally Kay ^116^, Liam Prestwood ^116^ and Dr Shavanthi Rajatileka ^116^

**Project administration, and Sequencing and analysis:**

Dr Joshua Quick ^43^

**Project administration, and Software and analysis tools:**

Radoslaw Poplawski ^43^

**Samples and logistics, and Sequencing and analysis:**

Dr Nicola Reynolds ^8^, Andrew Mack ^11^, Dr Arthur Morriss ^11^, Thomas Whalley ^11^, Bindi Patel ^12^, Dr Iliana Georgana ^24^, Dr Myra Hosmillo ^24^, Malte L Pinckert ^24^, Dr Joanne Stockton ^43^, Dr John H Henderson ^65^, Amy Hollis ^65^, Dr William Stanley ^65^, Dr Wen C Yew ^65^, Dr Richard Myers ^72^, Dr Alicia Thornton ^72^, Alexander Adams ^74^, Tara Annett ^74^, Dr Hibo Asad ^74^, Alec Birchley ^74^, Jason Coombes ^74^, Johnathan M Evans ^74^, Laia Fina ^74^, Bree Gatica-Wilcox ^74^, Lauren Gilbert ^74^, Lee Graham ^74^, Jessica Hey ^74^, Ember Hilvers ^74^, Sophie Jones ^74^, Hannah Jones ^74^, Sara Kumziene-Summerhayes ^74^, Dr Caoimhe McKerr ^74^, Jessica Powell ^74^, Georgia Pugh ^74^, Sarah Taylor ^74^, Alexander J Trotter ^75^, Charlotte A Williams ^96^, Leanne M Kermack ^102^, Benjamin H Foulkes ^109^, Marta Gallis ^109^, Hailey R Hornsby ^109^, Stavroula F Louka ^109^, Dr Manoj Pohare ^109^, Paige Wolverson ^109^, Peijun Zhang ^109^, George MacIntyre-Cockett ^115^, Amy Trebes ^115^, Dr Robin J Moll ^116^, Lynne Ferguson ^117^, Dr Emily J Goldstein ^117^, Dr Alasdair Maclean ^117^ and Dr Rachael Tomb ^117^

**Samples and logistics, and Software and analysis tools:**

Dr Igor Starinskij ^53^

**Sequencing and analysis, and Software and analysis tools:**

Laura Thomson ^5^, Joel Southgate ^11, 74^, Dr Moritz UG Kraemer ^23^, Dr Jayna Raghwani ^23^, Dr Alex E Zarebski ^23^, Olivia Boyd ^39^, Lily Geidelberg ^39^, Dr Chris J Illingworth ^52^, Dr Chris Jackson ^52^, Dr David Pascall ^52^, Dr Sreenu Vattipally ^53^, Timothy M Freeman ^109^, Dr Sharon N Hsu ^109^, Dr Benjamin B Lindsey ^109^, Dr Keith James ^116^, Kevin Lewis ^116^, Gerry Tonkin-Hill ^116^ and Dr Jaime M Tovar-Corona ^116^

**Sequencing and analysis, and Visualisation:**

MacGregor Cox ^20^

**Software and analysis tools, and Visualisation:**

Dr Khalil Abudahab ^14, 116^, Mirko Menegazzo ^14^, Ben EW Taylor MEng ^14, 116^, Dr Corin A Yeats ^14^, Afrida Mukaddas ^53^, Derek W Wright ^53^, Dr Leonardo de Oliveira Martins ^75^, Dr Rachel Colquhoun ^104^, Verity Hill ^104^, Dr Ben Jackson ^104^, Dr JT McCrone ^104^, Dr Nathan Medd ^104^, Dr Emily Scher ^104^ and Jon-Paul Keatley ^116^

**Leadership and supervision:**

Dr Tanya Curran ^3^, Dr Sian Morgan ^10^, Prof Patrick Maxwell ^20^, Prof Ken Smith ^20^, Dr Sahar Eldirdiri ^21^, Anita Kenyon ^21^, Prof Alison H Holmes ^38, 57^, Dr James R Price ^38, 57^, Dr Tim Wyatt ^69^, Dr Alison E Mather ^75^, Dr Timofey Skvortsov ^77^ and Prof John A Hartley ^96^

**Metadata curation:**

Prof Martyn Guest ^11^, Dr Christine Kitchen ^11^, Dr Ian Merrick ^11^, Robert Munn ^11^, Dr Beatrice Bertolusso ^33^, Dr Jessica Lynch ^33^, Dr Gabrielle Vernet ^33^, Stuart Kirk ^34^, Dr Elizabeth Wastnedge ^56^, Dr Rachael Stanley ^58^, Giles Idle ^64^, Dr Declan T Bradley ^69, 77^, Dr Jennifer Poyner ^79^ and Matilde Mori ^110^

**Project administration:**

Owen Jones ^11^, Victoria Wright ^18^, Ellena Brooks ^20^, Carol M Churcher ^20^, Mireille Fragakis ^20^, Dr Katerina Galai ^20, 70^, Dr Andrew Jermy ^20^, Sarah Judges ^20^, Georgina M McManus ^20^, Kim S Smith ^20^, Dr Elaine Westwick ^20^, Dr Stephen W Attwood ^23^, Dr Frances Bolt ^38, 57^, Dr Alisha Davies ^74^, Elen De Lacy ^74^, Fatima Downing ^74^, Sue Edwards ^74^, Lizzie Meadows ^75^, Sarah Jeremiah ^97^, Dr Nikki Smith ^109^ and Luke Foulser ^116^

**Samples and logistics:**

Dr Themoula Charalampous ^12, 46^, Amita Patel ^12^, Dr Louise Berry ^15^, Dr Tim Boswell ^15^, Dr Vicki M Fleming ^15^, Dr Hannah C Howson-Wells ^15^, Dr Amelia Joseph ^15^, Manjinder Khakh ^15^, Dr Michelle M Lister ^15^, Paul W Bird ^16^, Karlie Fallon ^16^, Thomas Helmer ^16^, Dr Claire L McMurray ^16^, Mina Odedra ^16^, Jessica Shaw ^16^, Dr Julian W Tang ^16^, Nicholas J Willford ^16^, Victoria Blakey ^17^, Dr Veena Raviprakash ^17^, Nicola Sheriff ^17^, Lesley-Anne Williams ^17^, Theresa Feltwell ^20^, Dr Luke Bedford ^26^, Dr James S Cargill ^27^, Warwick Hughes ^27^, Dr Jonathan Moore ^28^, Susanne Stonehouse ^28^, Laura Atkinson ^29^, Jack CD Lee ^29^, Dr Divya Shah ^29^, Adela Alcolea-Medina ^32, 112^, Natasha Ohemeng-Kumi ^32, 112^, John Ramble ^32, 112^, Jasveen Sehmi ^32, 112^, Dr Rebecca Williams ^33^, Wendy Chatterton ^34^, Monika Pusok ^34^, William Everson ^37^, Anibolina Castigador ^44^, Emily Macnaughton ^44^, Dr Kate El Bouzidi ^45^, Dr Temi Lampejo ^45^, Dr Malur Sudhanva ^45^, Cassie Breen ^47^, Dr Graciela Sluga ^48^, Dr Shazaad SY Ahmad ^49, 70^, Dr Ryan P George ^49^, Dr Nicholas W Machin ^49, 70^, Debbie Binns ^50^, Victoria James ^50^, Dr Rachel Blacow ^55^, Dr Lindsay Coupland ^58^, Dr Louise Smith ^59^, Dr Edward Barton ^60^, Debra Padgett ^60^, Garren Scott ^60^, Dr Aidan Cross ^61^, Dr Mariyam Mirfenderesky ^61^, Jane Greenaway ^62^, Kevin Cole ^64^, Phillip Clarke ^67^, Nichola Duckworth ^67^, Sarah Walsh ^67^, Kelly Bicknell ^68^, Robert Impey ^68^, Dr Sarah Wyllie ^68^, Richard Hopes ^70^, Dr Chloe Bishop ^72^, Dr Vicki Chalker ^72^, Dr Ian Harrison ^72^, Laura Gifford ^74^, Dr Zoltan Molnar ^77^, Dr Cressida Auckland ^79^, Dr Cariad Evans ^85, 109^, Dr Kate Johnson ^85, 109^, Dr David G Partridge ^85, 109^, Dr Mohammad Raza ^85, 109^, Paul Baker ^86^, Prof Stephen Bonner ^86^, Sarah Essex ^86^, Leanne J Murray ^86^, Andrew I Lawton ^87^, Dr Shirelle Burton-Fanning ^89^, Dr Brendan AI Payne ^89^, Dr Sheila Waugh ^89^, Andrea N Gomes ^91^, Maimuna Kimuli ^91^, Darren R Murray ^91^, Paula Ashfield ^92^, Dr Donald Dobie ^92^, Dr Fiona Ashford ^93^, Dr Angus Best ^93^, Dr Liam Crawford ^93^, Dr Nicola Cumley ^93^, Dr Megan Mayhew ^93^, Dr Oliver Megram ^93^, Dr Jeremy Mirza ^93^, Dr Emma Moles-Garcia ^93^, Dr Benita Percival ^93^, Megan Driscoll ^96^, Leah Ensell ^96^, Dr Helen L Lowe ^96^, Laurentiu Maftei ^96^, Matteo Mondani ^96^, Nicola J Chaloner ^99^, Benjamin J Cogger ^99^, Lisa J Easton ^99^, Hannah Huckson ^99^, Jonathan Lewis ^99^, Sarah Lowdon ^99^, Cassandra S Malone ^99^, Florence Munemo ^99^, Manasa Mutingwende ^99^, Roberto Nicodemi ^99^, Olga Podplomyk ^99^, Thomas Somassa ^99^, Dr Andrew Beggs ^100^, Dr Alex Richter ^100^, Claire Cormie ^102^, Joana Dias ^102^, Sally Forrest ^102^, Dr Ellen E Higginson ^102^, Mailis Maes ^102^, Jamie Young ^102^, Dr Rose K Davidson ^103^, Kathryn A Jackson ^107^, Dr Lance Turtle ^107^, Dr Alexander J Keeley ^109^, Prof Jonathan Ball ^113^, Timothy Byaruhanga ^113^, Dr Joseph G Chappell ^113^, Jayasree Dey ^113^, Jack D Hill ^113^, Emily J Park ^113^, Arezou Fanaie ^114^, Rachel A Hilson ^114^, Geraldine Yaze ^114^ and Stephanie Lo ^116^

**Sequencing and analysis:**

Safiah Afifi ^10^, Robert Beer ^10^, Joshua Maksimovic ^10^, Kathryn McCluggage ^10^, Karla Spellman ^10^, Catherine Bresner ^11^, William Fuller ^11^, Dr Angela Marchbank ^11^, Trudy Workman ^11^, Dr Ekaterina Shelest ^13, 81^, Dr Johnny Debebe ^18^, Dr Fei Sang ^18^, Dr Marina Escalera Zamudio ^23^, Dr Sarah Francois ^23^, Bernardo Gutierrez ^23^, Dr Tetyana I Vasylyeva ^23^, Dr Flavia Flaviani ^31^, Dr Manon Ragonnet-Cronin ^39^, Dr Katherine L Smollett ^42^, Alice Broos ^53^, Daniel Mair ^53^, Jenna Nichols ^53^, Dr Kyriaki Nomikou ^53^, Dr Lily Tong ^53^, Ioulia Tsatsani ^53^, Prof Sarah O'Brien ^54^, Prof Steven Rushton ^54^, Dr Roy Sanderson ^54^, Dr Jon Perkins ^55^, Seb Cotton ^56^, Abbie Gallagher ^56^, Dr Elias Allara ^70, 102^, Clare Pearson ^70, 102^, Dr David Bibby ^72^, Dr Gavin Dabrera ^72^, Dr Nicholas Ellaby ^72^, Dr Eileen Gallagher ^72^, Dr Jonathan Hubb ^72^, Dr Angie Lackenby ^72^, Dr David Lee ^72^, Nikos Manesis ^72^, Dr Tamyo Mbisa ^72^, Dr Steven Platt ^72^, Katherine A Twohig ^72^, Dr Mari Morgan ^74^, Alp Aydin ^75^, David J Baker ^75^, Dr Ebenezer Foster-Nyarko ^75^, Dr Sophie J Prosolek ^75^, Steven Rudder ^75^, Chris Baxter ^77^, Sílvia F Carvalho ^77^, Dr Deborah Lavin ^77^, Dr Arun Mariappan ^77^, Dr Clara Radulescu ^77^, Dr Aditi Singh ^77^, Miao Tang ^77^, Helen Morcrette ^79^, Nadua Bayzid ^96^, Marius Cotic ^96^, Dr Carlos E Balcazar ^104^, Dr Michael D Gallagher ^104^, Dr Daniel Maloney ^104^, Thomas D Stanton ^104^, Dr Kathleen A Williamson ^104^, Dr Robin Manley ^105^, Michelle L Michelsen ^105^, Dr Christine M Sambles ^105^, Dr David J Studholme ^105^, Joanna Warwick-Dugdale ^105^, Richard Eccles ^107^, Matthew Gemmell ^107^, Dr Richard Gregory ^107^, Dr Margaret Hughes ^107^, Charlotte Nelson ^107^, Dr Lucille Rainbow ^107^, Dr Edith E Vamos ^107^, Hermione J Webster ^107^, Dr Mark Whitehead ^107^, Claudia Wierzbicki ^107^, Dr Adrienn Angyal ^109^, Dr Luke R Green ^109^, Dr Max Whiteley ^109^, Emma Betteridge ^116^, Dr Iraad F Bronner ^116^, Ben W Farr ^116^, Scott Goodwin ^116^, Dr Stefanie V Lensing ^116^, Shane A McCarthy ^116, 102^, Dr Michael A Quail ^116^, Diana Rajan ^116^, Dr Nicholas M Redshaw ^116^, Carol Scott ^116^, Lesley Shirley ^116^ and Scott AJ Thurston ^116^

**Software and analysis tools:**

Dr Will Rowe ^43^, Amy Gaskin ^74^, Dr Thanh Le-Viet ^75^, James Bonfield ^116^, Jennifier Liddle ^116^ and Andrew Whitwham ^116^

**1** Barking, Havering and Redbridge University Hospitals NHS Trust, **2** Barts Health NHS Trust, **3** Belfast Health & Social Care Trust, **4** Betsi Cadwaladr University Health Board, **5** Big Data Institute, Nuffield Department of Medicine, University of Oxford, **6** Blackpool Teaching Hospitals NHS Foundation Trust, **7** Bournemouth University, **8** Cambridge Stem Cell Institute, University of Cambridge, **9** Cambridge University Hospitals NHS Foundation Trust, **10** Cardiff and Vale University Health Board, **11** Cardiff University, **12** Centre for Clinical Infection and Diagnostics Research, Department of Infectious Diseases, Guy's and St Thomas' NHS Foundation Trust, **13** Centre for Enzyme Innovation, University of Portsmouth, **14** Centre for Genomic Pathogen Surveillance, University of Oxford, **15** Clinical Microbiology Department, Queens Medical Centre, Nottingham University Hospitals NHS Trust, **16** Clinical Microbiology, University Hospitals of Leicester NHS Trust, **17** County Durham and Darlington NHS Foundation Trust, **18** Deep Seq, School of Life Sciences, Queens Medical Centre, University of Nottingham, **19** Department of Infectious Diseases and Microbiology, Cambridge University Hospitals NHS Foundation Trust, **20** Department of Medicine, University of Cambridge, **21** Department of Microbiology, Kettering General Hospital, **22** Department of Microbiology, South West London Pathology, **23** Department of Zoology, University of Oxford, **24** Division of Virology, Department of Pathology, University of Cambridge, **25** East Kent Hospitals University NHS Foundation Trust, **26** East Suffolk and North Essex NHS Foundation Trust, **27** East Sussex Healthcare NHS Trust**,** **28** Gateshead Health NHS Foundation Trust, **29** Great Ormond Street Hospital for Children NHS Foundation Trust, **30** Great Ormond Street Institute of Child Health (GOS ICH), University College London (UCL), **31** Guy's and St. Thomas’ Biomedical Research Centre, **32** Guy's and St. Thomas’ NHS Foundation Trust, **33** Hampshire Hospitals NHS Foundation Trust, **34** Health Services Laboratories, **35** Heartlands Hospital, Birmingham, **36** Hub for Biotechnology in the Built Environment, Northumbria University, **37** Hull University Teaching Hospitals NHS Trust, **38** Imperial College Healthcare NHS Trust, **39** Imperial College London, **40** Infection Care Group, St George’s University Hospitals NHS Foundation Trust, **41** Institute for Infection and Immunity, St George’s University of London, **42** Institute of Biodiversity, Animal Health & Comparative Medicine, **43** Institute of Microbiology and Infection, University of Birmingham, **44** Isle of Wight NHS Trust, **45** King's College Hospital NHS Foundation Trust, **46** King's College London, **47** Liverpool Clinical Laboratories, **48** Maidstone and Tunbridge Wells NHS Trust, **49** Manchester University NHS Foundation Trust, **50** Microbiology Department, Buckinghamshire Healthcare NHS Trust, **51** Microbiology, Royal Oldham Hospital, **52** MRC Biostatistics Unit, University of Cambridge, **53** MRC-University of Glasgow Centre for Virus Research, **54** Newcastle University, **55** NHS Greater Glasgow and Clyde, **56** NHS Lothian, **57** NIHR Health Protection Research Unit in HCAI and AMR, Imperial College London, **58** Norfolk and Norwich University Hospitals NHS Foundation Trust, **59** Norfolk County Council, **60** North Cumbria Integrated Care NHS Foundation Trust, **61** North Middlesex University Hospital NHS Trust, **62** North Tees and Hartlepool NHS Foundation Trust, **63** North West London Pathology, **64** Northumbria Healthcare NHS Foundation Trust, **65** Northumbria University, **66** NU-OMICS, Northumbria University, **67** Path Links, Northern Lincolnshire and Goole NHS Foundation Trust, **68** Portsmouth Hospitals University NHS Trust, **69** Public Health Agency, Northern Ireland, **70** Public Health England, **71** Public Health England, Cambridge, **72** Public Health England, Colindale, **73** Public Health Scotland, **74** Public Health Wales, **75** Quadram Institute Bioscience, **76** Queen Elizabeth Hospital, Birmingham, **77** Queen's University Belfast, **78** Royal Brompton and Harefield Hospitals, **79** Royal Devon and Exeter NHS Foundation Trust, **80** Royal Free London NHS Foundation Trust, **81** School of Biological Sciences, University of Portsmouth, **82** School of Health Sciences, University of Southampton, **83** School of Medicine, University of Southampton, **84** School of Pharmacy & Biomedical Sciences, University of Portsmouth, **85** Sheffield Teaching Hospitals NHS Foundation Trust, **86** South Tees Hospitals NHS Foundation Trust, **87** Southwest Pathology Services, **88** Swansea University, **89** The Newcastle upon Tyne Hospitals NHS Foundation Trust, **90** The Queen Elizabeth Hospital King's Lynn NHS Foundation Trust, **91** The Royal Marsden NHS Foundation Trust, **92** The Royal Wolverhampton NHS Trust, **93** Turnkey Laboratory, University of Birmingham, **94** University College London Division of Infection and Immunity**, 95** University College London Hospital Advanced Pathogen Diagnostics Unit**, 96** University College London Hospitals NHS Foundation Trust, **97** University Hospital Southampton NHS Foundation Trust, **98** University Hospitals Dorset NHS Foundation Trust, **99** University Hospitals Sussex NHS Foundation Trust, **100** University of Birmingham, **101** University of Brighton, **102** University of Cambridge, **103** University of East Anglia, **104** University of Edinburgh, **105** University of Exeter, **106** University of Kent, **107** University of Liverpool, **108** University of Oxford, **109** University of Sheffield, **110** University of Southampton, **111** University of St Andrews, **112** Viapath, Guy's and St Thomas' NHS Foundation Trust, and King's College Hospital NHS Foundation Trust, **113** Virology, School of Life Sciences, Queens Medical Centre, University of Nottingham, **114** Watford General Hospital, **115** Wellcome Centre for Human Genetics, Nuffield Department of Medicine, University of Oxford, **116** Wellcome Sanger Institute, **117** West of Scotland Specialist Virology Centre, NHS Greater Glasgow and Clyde, **118** Whittington Health NHS Trust
