# Supplementary material for: Tracking SARS-CoV-2 mutations and variants through the COG-UK-Mutation Explorer
Source: Virus Evol. 2022 Mar 18;8(1):veac023. doi: 10.1093/ve/veac023 (PMC9037374; doi:10.1093/ve/veac023)
Supplement: veac023_Supp [file veac023_supp.zip › SI_COG-UK-Mutation_Explorer__A_web_Application_for_Tracking_SARS-CoV-2_Mutations.docx]

**Tracking SARS-CoV-2 mutations & variants through the COG-UK-Mutation Explorer**

Derek W Wright^1^, William T Harvey^1^, Joseph Hughes^1^, MacGregor Cox^2^, Thomas P Peacock^3^, Rachel Colquhoun^4^, Ben Jackson^4^, Richard Orton^1^, Morten Nielsen^6^, Nienyun Sharon Hsu^9^, The COVID-19 Genomics UK (COG-UK) consortium^5^, Ewan M Harrison^2,7,8^, Thushan I de Silva^9^, Andrew Rambaut^4^, Sharon J Peacock^2^, David L Robertson^1#^, Alessandro M Carabelli^2#^

^1^MRC-University of Glasgow Centre for Virus Research, University of Glasgow, Glasgow, UK

^2^Department of Medicine, University of Cambridge, Cambridge, UK

^3^Department of Infectious Disease, St Mary’s Medical School, Imperial College London, UK

^4^Institute of Evolutionary Biology, University of Edinburgh, Edinburgh, UK

^5^https://www.cogconsortium.uk. Full list of consortium names and affiliations are in Appendix 1

^6^Universidad Nacional de San Martin, Argentina

^7^Wellcome Sanger Institute, Hinxton, UK

^8^Department of Public Health and Primary Care, University of Cambridge, Cambridge, UK

^9^The Florey Institute for Host-Pathogen Interactions and Department of Infection, Immunity and Cardiovascular Disease, Medical School, University of Sheffield, Sheffield, UK

^#^Contact: [david.l.robertson@glasgow.ac.uk](mailto:david.l.robertson@glasgow.ac.uk) and [amc257@medsch.cam.ac.uk](mailto:amc257@medsch.cam.ac.uk)


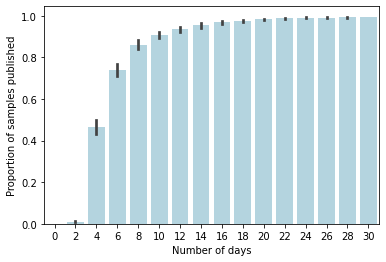


**Figure S1:** The histogram shows the proportion of samples published for a specific date (n=100) over the following number of days. Error bars show +/- 1 standard deviation.

**
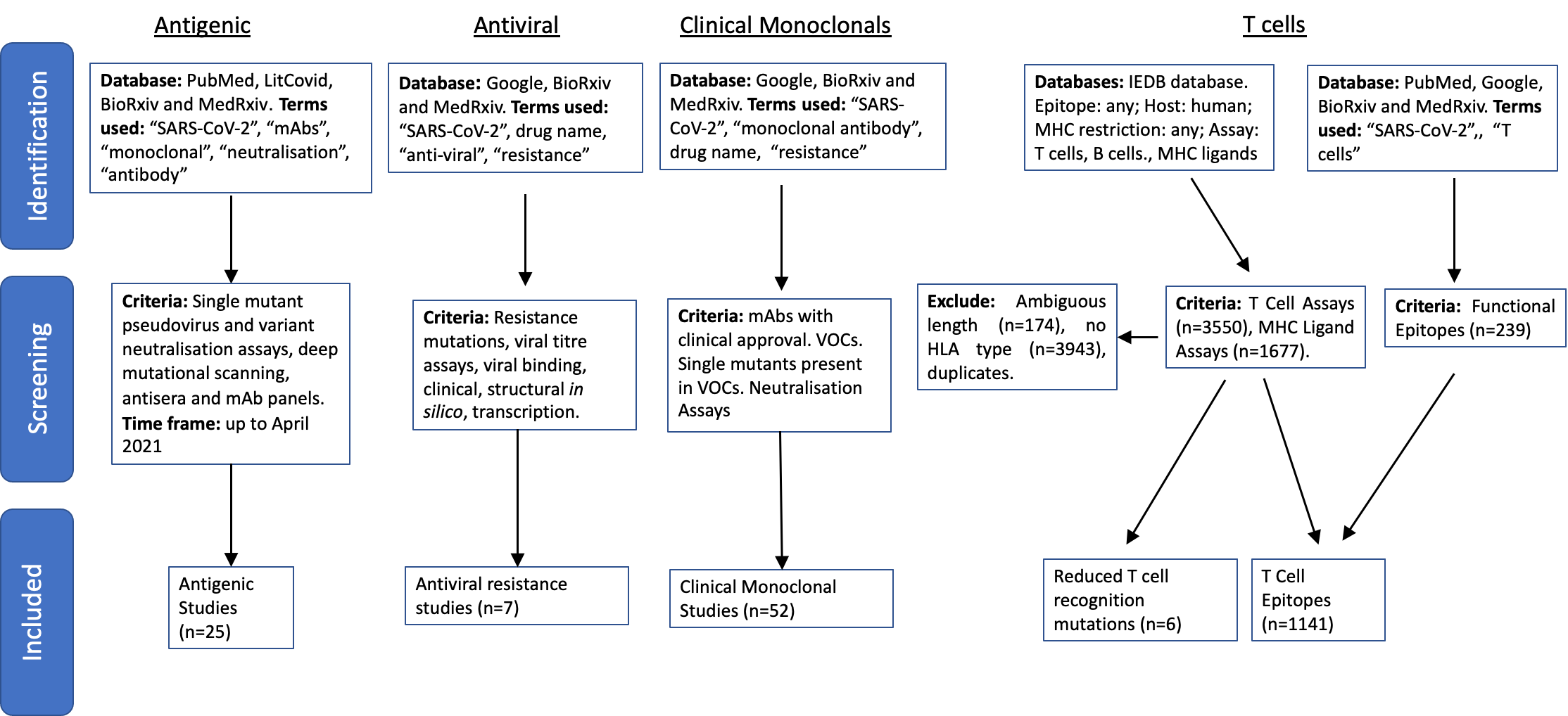
**

**Figure S2:** Flow of information through the different phases of the literature review conducted on four different areas: antigenic and T-cells; antivirals and monoclonals mAbs. The flow highlights the different databases used to search for specific terms, the criteria used to screen the literature and the inclusion results for each area.
